# Structure–Performance Relationships in Metal–Organic Framework Mg_2_(olz) Catalysts for CO_2_ Adsorption and Hydrogenation to Methanol

**DOI:** 10.1002/cssc.71202

**Published:** 2026-11-05

**Authors:** Anna Liutkova, Fabio André Peixoto Esteves, Anastasia Molokova, Marianela López Romero, Davide Salusso, Andrea Testino, Emiliya Poghosyan, Marco Ranocchiari

**Affiliations:** ^1^ Center for Energy and Environmental Sciences Paul Scherrer Institute PSI Villigen Switzerland; ^2^ Department of Chemistry and Applied Biosciences ETH Zürich Zurich Switzerland; ^3^ ESRF, The European Synchrotron Grenoble France; ^4^ Center for Life Sciences Paul Scherrer Institute PSI Villigen Switzerland

**Keywords:** CO_2_ adsorption, CO_2_ hydrogenation, in situ spectroscopy, metal–organic frameworks, methanol synthesis

## Abstract

We investigated a Cu–Zn‐modified Mg_2_(olz) catalyst for carbon dioxide (CO_2_) hydrogenation to methanol under low CO_2_ partial pressures, relevant to dilute CO_2_ streams in industrial gas treatment and carbon capture. The catalyst was prepared via wet impregnation of presynthesized Cu–Zn species into the metal‐organic framework (MOF). Batch tests under mild temperatures and low CO_2_ concentration showed that the MOF‐based system achieved higher methanol yields than a commercial catalyst at 150 °C. Step‐response experiments in a plug‐flow reactor revealed that the MOF retained a significant amount of CO_2_ under high‐pressure and dynamic conditions. In situ diffuse reflectance infrared Fourier transform spectroscopy identified methanol‐related surface intermediates, while ex situ postreaction ^1^H nuclear magnetic resonance spectroscopy supported the formation and retention of methanol and oxygenated species in the MOF‐based catalyst. X‐ray absorption spectroscopy showed partial reduction of oxygenated Cu species during reaction, while in situ X‐ray diffraction demonstrated structural stability of the Mg_2_(olz) MOF framework. These results indicate that Mg_2_(olz)‐based materials combine CO_2_ adsorption capacity with catalytic functionality. Adsorption and catalytic functions coexist rather than being directly coupled. The material exhibits methanol productivity at low temperature in the presence of a solvent phase and remains structurally stable under the investigated catalytic conditions.

## Graphical abstract

A Cu(Zn)/Mg_2_(olz) metal–organic framework catalyst was investigated under liquid‐ and gas‐phase conditions. Higher methanol productivity than a commercial Cu/ZnO/Al_2_O_3_ catalyst was achieved at 150 °C. Combined spectroscopy and transient experiments reveal carbon dioxide uptake, growth of methanol‐related intermediates, framework stability, and the importance of the solvent for methanol release.

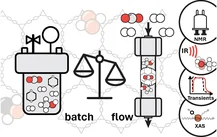

## 1 Introduction

The development of efficient catalysts for carbon dioxide (CO_2_)‐to‐methanol conversion is critical for mitigating CO_2_ emissions [1] while enabling sustainable methanol production. Methanol is a key chemical feedstock and an emerging energy carrier, making its direct synthesis from CO_2_ a highly relevant research area [2, 3]. In this context, the concentration of CO_2_ in the feed must also be carefully considered during catalyst design. Catalytic materials capable of capturing and converting dilute CO_2_ streams are attractive for integration into postcombustion processes and industrial gas cleanup [4]. Additionally, traditional catalysts used in the industry for syngas mixtures typically require high operating temperatures, which increases the energy demand [5]. Lowering the reaction temperature without compromising methanol productivity remains a major challenge in the development of next‐generation catalysts.

Metal‐organic frameworks [6, 7] (MOFs) provide a promising platform for CO_2_ conversion given their high CO_2_ adsorption capacity [8], tunable porosity, and the ability to host or support catalytically active metal species [9]. MOFs such as zeolitic imidazolate frameworks (ZIF‐8, ZIF‐67), UiO‐66, and MOF‐74 have been studied for catalytic CO_2_ hydrogenation, both in gas‐phase and in batch liquid‐phase systems [9, 10, 11, 12, 13]. Gas‐phase CO_2_ hydrogenation over Cu nanoparticles encapsulated in ZIF‐8 has shown enhanced methanol selectivity and catalyst stability compared to traditional Cu/ZnO/Al_2_O_3_ catalysts, in part attributed to spatial organization of the active phase within the porous framework [14]. In UiO‐66, the formation of copper–zirconia nanodomains enabled >70% methanol selectivity at 180–210 °C [15], and further engineering of interfacial Cu–ZrO_2_ sites within MOFs improved the durability and selectivity of the catalyst [16]. Among MOF candidates, the MOF‐74 family emerges as a particularly promising structure, known for its CO_2_ adsorption capacity and tunability [8, 17, 18]. MOF‐74 itself, also known as M_2_(dobdc), contains a framework with a high density of coordinatively unsaturated metal centers that contribute to its strong adsorption properties and modular synthesis [19]. MOF‐74 and its expanded analogs with longer linkers, such as IRMOF‐74(n), where n stands for the number of aromatic rings in a linker, offer tunable pore environments that can be adapted for catalysis or adsorption [20, 21]. Mg‐MOF‐74, in particular, shows exceptional CO_2_ uptake, with values ~8 mmol/g at 1 bar and 298 K [22] due to strong binding at the Mg^2+^ open metal sites [23]. Its olsalazine‐functionalized analog Mg_2_(olz) gives a larger‐pore version, improving diffusion and adsorption [18]. Mixed‐metal Ni–Mg MOF‐74‐templated systems have been evaluated for gas‐phase CO_2_ hydrogenation and were found to be catalytically active [24], while Cu–Zn MOF‐74 catalysts obtained by mechanochemical amorphization showed better metal dispersion and improved methanol selectivity compared to conventionally prepared catalysts [25]. Batch‐phase CO_2_ hydrogenation using MOF‐based [26] and covalent organic framework (COF)‐based systems [27] has recently gained attention, enabling mechanistic studies under structurally well‐defined conditions [13, 28, 29]. For example, Fellenberg et al. demonstrated that COF functionalized with atomically dispersed Ru sites catalyzed the liquid‐phase hydrogenation of CO_2_ to formic acid under mild conditions, with operando X‐ray absorption (XAS) resolving the local environment of active species [27]. These results suggest that these framework materials can work in both batch‐liquid and continuous flow gas‐phase CO_2_ valorization.

The integration of external metal species with MOFs is widely described as a strategy to combine complementary functionalities rather than relying solely on pore confinement [30, 31, 32, 33]. MOFs can stabilize these metal species, mitigate aggregation, and enable catalytic effects arising from the metal–framework interface [34, 35]. Depending on the synthesis route, metal species may be located inside pores or on the external surface, with catalytic behavior governed by host–guest interactions and interfacial effects rather than strict confinement [35, 36, 37]. Accordingly, MOF‐based materials functionalized with metals are best described as multifunctional systems, where the framework provides adsorption capacity and chemical versatility, while the external metal species supply the primary catalytic function [38].

Previous studies show that MOF‐74‐type materials combine high CO_2_ uptake with porous frameworks that can support Cu–Zn species participating in methanol formation at moderate temperatures. This makes them relevant for conditions where CO_2_ is present in diluted feeds and where the reaction can be carried out at lower temperatures than in conventional Cu/ZnO/Al_2_O_3_ systems. Such multifunctional materials are of growing interest for integrating CO_2_ capture with catalytic conversion. In this work, we investigate Mg_2_(olz) modified with Cu–Zn species to understand how CO_2_ adsorption, methanol formation, and structural changes evolve under low‐CO_2_ and low‐temperature conditions.

## 2 Materials and Methods

The MOF‐74 framework with olsalazine linkers Mg_2_(olz) was synthesized solvothermally according to a literature procedure [17, 18] and later modified with Cu–Zn species, denoted as Cu(Zn). The scheme of the synthesis is shown in Figure S1a. The materials were characterized using ^1^H nuclear magnetic resonance (NMR) spectroscopy, powder X‐ray diffraction (XRD), attenuated total reflectance infrared spectroscopy (ATR‐IR), transmission electron microscopy (TEM) and scanning transmission electron microscopy coupled with energy‐dispersive X‐ray spectroscopy (STEM‐EDX), inductively coupled plasma optical emission spectroscopy (ICP‐OES), thermogravimetric analysis coupled with mass spectrometry (TGA‐MS), N_2_ physisorption, H_2_ chemisorption, in situ diffuse reflectance infrared Fourier transform spectroscopy (DRIFTS), and XAS spectroscopy coupled with XRD. An industrial Cu/ZnO/Al_2_O_3_ [39] catalyst (CZA; product number: 45776; lot number I06Z036) containing 10.1 wt.% Al_2_O_3_, 63.5 wt.% CuO, 24.7 wt.% ZnO, and 1.3 wt.% MgO was purchased from Alfa Aesar. The detailed description of the methods and the synthesis are given in the Supporting Information.

## 3 Results and Discussion

First, we characterized the properties of the synthesized material. The ^1^H NMR spectroscopy, XRD, and ATR‐IR indicated that we obtained the crystalline MOF with olsalazine linkers (Figures S1b,c and S2a). Although the samples were transferred from air‐free storage and measured under ambient conditions, the wide IR band at ~3400 cm^−1^ in the pristine Mg_2_(olz) MOF is attributed to rapid H_2_O adsorption from air at open metal sites. ^1^H NMR spectroscopy demonstrated the absence of residual dimethylformamide expected signals at 8.0 and 2.9 ppm) and ethanol (3.6 and 1.2 ppm) after activation under vacuum (Figure S1b). TEM revealed that the MOF has a sheet‐like morphology of 100–150 nm in diameter (Figure 1), which can provide a relatively large surface area for fast diffusion channels and anchoring of external species. From the line scan for atomic spacing identification, we estimated an interplanar distance of 21 Å. This number correlates with the theoretical size of the Mg_2_(olz) MOF pore, 27 Å [18]. The MOF possesses a high surface area of 2620 m^2^/g and a pore volume of 1.5 cm^3^/g from the N_2_ physisorption measurements. The physico–chemical properties of the Mg_2_(olz) and Mg_2_(olz)‐based catalyst are summarized in Table 1, while nitrogen sorption isotherms are reported in Figure S3.

**FIGURE 1:**
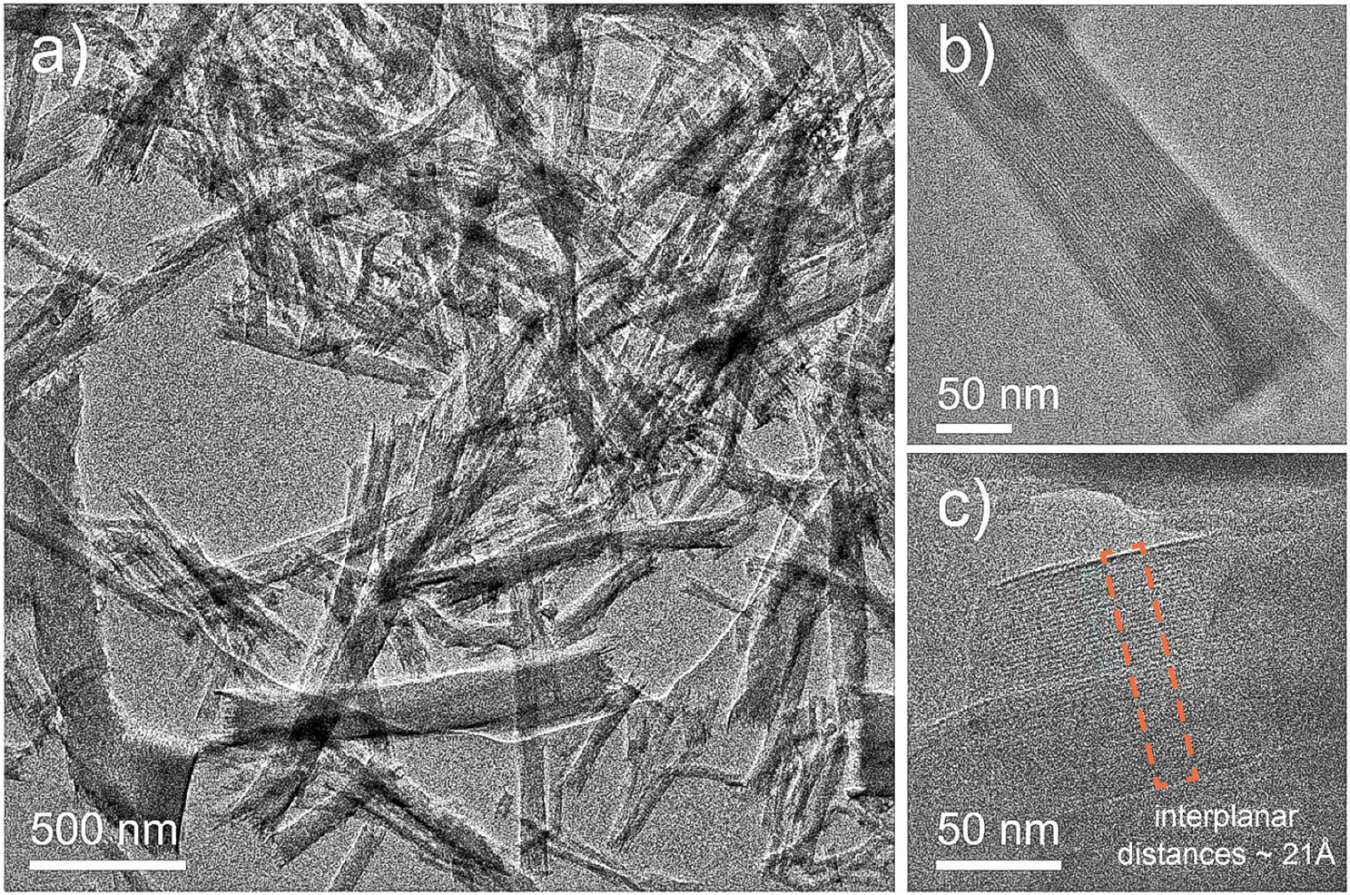
TEM images of the Mg_2_(olz) at different magnifications (a–c), showing sheet‐like, elongated structures. The highlighted orange area in image (c) corresponds to the region where an intensity histogram was taken for atomic spacing identification.

**TABLE 1:** N_2_ physisorption results and chemical composition of MOF‐based materials.

Sample	***S***_**BET,**_ **m** ^**2**^ **/g**	**V, cm** ^**3**^ **/g**	Mg, wt.%	Cu, wt.%	Zn, wt.%
Mg_2_(olz)	2620	1.5	11.2	—	—
Cu(Zn)/Mg_2_(olz)	1470	0.6	10.3	4.3	0.2

The stability of the Mg_2_(olz) framework is crucial for CO_2_ hydrogenation, which operates at elevated temperatures and pressures. To assess this, we performed in situ TGA‐MS in air and H_2_‐containing atmospheres (Figure 2). In air, the framework did not show evidence of degradation up to ~250 °C (Figure 2a). The derivative thermogravimetry (DTG) curve and MS traces for *m/z* = 12, 18, 44 (corresponding to carbon, water, and CO_2_, respectively) showed three events: removal of volatile adsorbates (>200 °C) and a two‐step thermal decomposition at 250–400 °C and at 400–500 °C (Figure 2a,c) [40]. The residual mass corresponded to 18.9 wt% MgO (11.4 wt% Mg), in good agreement with the ICP data (11.2 wt% Mg) [41]. Under an H_2_‐containing atmosphere, decomposition started earlier (~200 °C) and proceeded gradually up to ~700 °C. At *T* > 800 °C, CO (not shown) was formed, as a consequence of the reaction between residual water, CO_2_, and C, giving a significant signal at *m/z* = 12. Water signals came from initial humidity (*T* < 150 °C) and from several solid–gas reactions through the entire thermal treatment.

**FIGURE 2:**
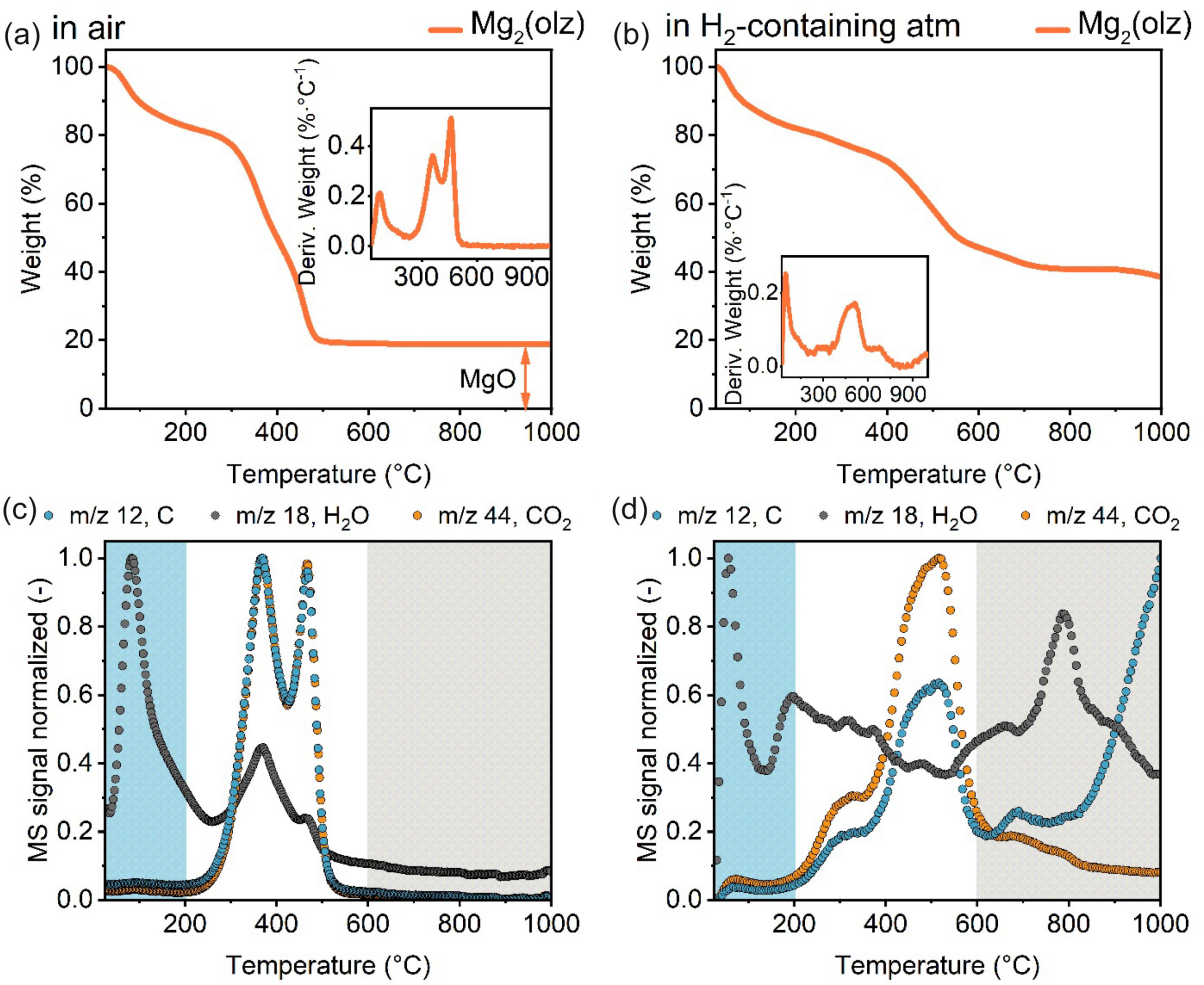
TGA‐MS of the Mg_2_(olz) in air (a,c) and in a H_2_‐containing atmosphere (b,d). DTG curve is an inset in (a,b). MS signal *m/z* 12 corresponds to carbon, 18 to water, and 44 to CO_2_. Conditions: 19 mL/min He + 1 mL/min H_2_ or 20 mL/min air, 50–1000 °C, ambient pressure, heating rate 5 °C/min. The blue highlight in (c,d) corresponds to the removal of the volatile adsorbates such as water and solvents (0–200 °C), white area (200–400 °C), decomposition of the MOF, and gray highlight (600–1000 °C) corresponds to residues.

Overall, this behavior suggests the complex decomposition of the linker that starts at 200 °C and accumulation of carbonaceous residues, not completely gasified at 1000 °C, in contrast to the complete combustion pathway observed in air, where the CO_2_ release is sharper and no significant residue remains. This demonstrates that Mg_2_(olz) is stable in the temperature range relevant for CO_2_ hydrogenation, while decomposition above ~250 °C in air and ~200 °C in H_2_ marks the onset of framework degradation, which lies above the operational range (150–200 °C).

Having established the stability of the Mg_2_(olz) framework, we next synthesized the Cu(Zn)/Mg_2_(olz) catalyst. To prepare the Cu(Zn)/Mg_2_(olz) catalyst, presynthesized [42]Cu(Zn) species were introduced into Mg_2_(olz) by wet impregnation in dry acetonitrile inside a glovebox (Sigma–Aldrich, 99.8%; synthesis scheme in Figure S4b). Acetonitrile was chosen for its easy removal under vacuum at 200 °C and its weak interaction with open metal sites. The Cu(Zn) species were characterized prior to loading by XRD (Figure S4a) and H_2_‐TPR (Figure S4c), showing Cu reduction at 175 °C and possible Zn reduction at ~370 °C. After incorporation, STEM–EDX demonstrated that Cu‐containing species are located in close proximity to the MOF surface (Figure S4d–f). The resulting Cu(Zn)/Mg_2_(olz) retained its characteristic crystalline structure, while its BET surface area decreased from 2620 to 1470 m^2^/g and micropore volume from 1.4 to 0.6 cm^3^/g (Figure S3; Tables 1 and S1). This reduction may result from partial pore obstruction by Cu(Zn) species and residual acetonitrile from impregnation (Figure S2), although partial framework degradation cannot be excluded. TEM images (Figures 3 and S5) showed preserved sheet‐like morphology. The observed Cu(Zn) features are predominantly aggregated, irregular assemblies with projected dimensions above 250 nm (0.25–1 µm), as confirmed by EDX, rather than discrete nanoparticles. No evidence for incorporation inside the pores is observed by the methods available, indicating that deposition occurs mainly on the external surface of the MOF crystals, which implies that catalytic behavior arises from externally accessible Cu(Zn) domains rather than confined active sites. Given the aggregated morphology, a conventional nanoparticle size distribution cannot be derived from the present TEM data.

**FIGURE 3:**
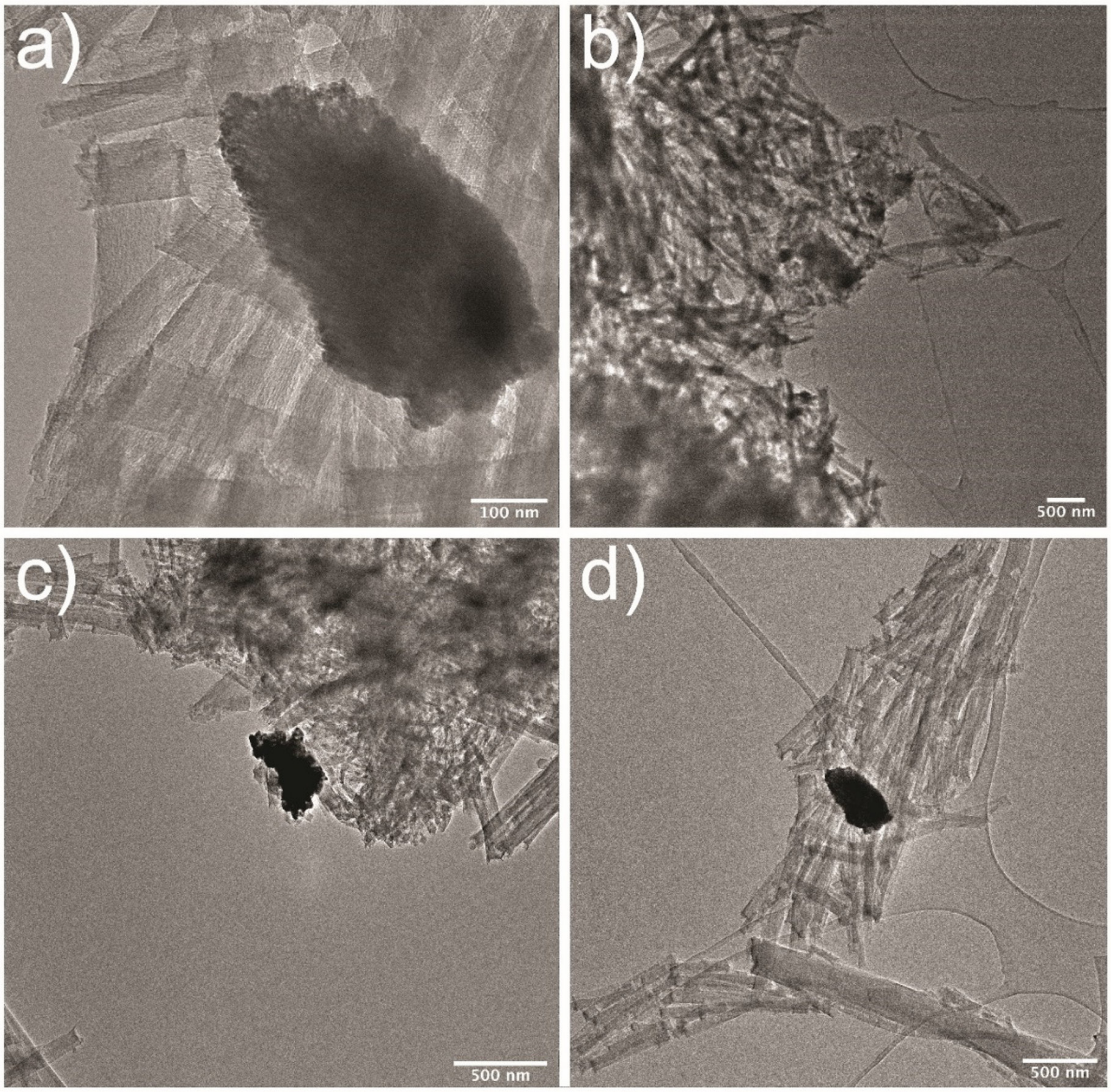
TEM images of the Cu(Zn)/Mg_2_(olz) catalyst at various magnifications (a–d). The Cu(Zn) species form large, dark particles which are colocalized with the sheet‐like Mg_2_(olz) structures.

The Cu(Zn)/Mg_2_(olz) catalyst was tested for CO_2_ hydrogenation to methanol in a stainless‐steel autoclave at 150 and 180 °C, 52 bar total pressure, and a CO_2_:H_2_ feed ratio of 1:26, simulating low CO_2_ partial pressures. Prior to the test, the catalyst was suspended in dioxane, which served as an inert, aprotic solvent to ensure the homogeneous distribution of the MOF‐based material and effective contact with the gas phase (Figure S6). The schematic of the experiment is shown in Figure 4a. For comparison, a commercial CZA catalyst containing the same amount of (Cu + Zn) was tested under identical conditions. We note that, as an oxide powder, CZA does not form a suspension to the same extent as the MOF‐based material; however, it was included as a benchmark catalyst to provide a reference point within the Cu/Zn methanol synthesis class. Owing to the fundamentally different material classes, this comparison cannot be considered fully identical but serves as a practical activity benchmark under the same reactor conditions. The results are provided in Figure 4b.

**FIGURE 4:**
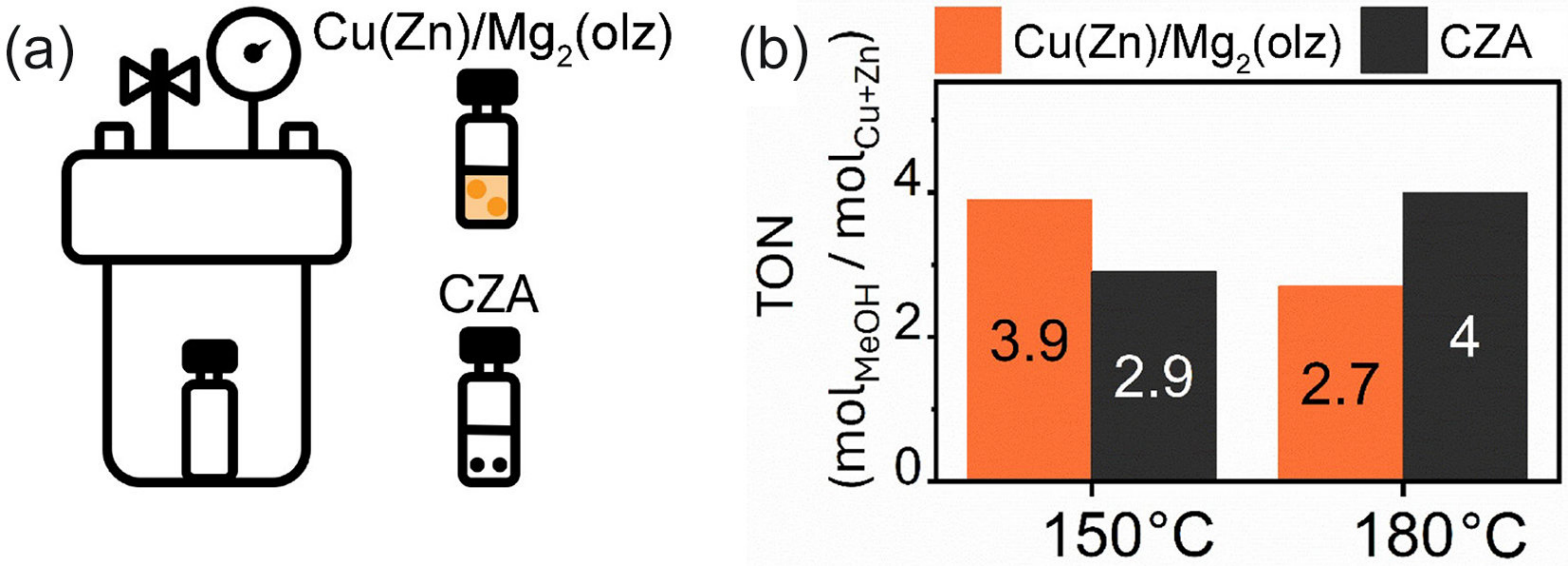
(a) Schematic description of the batch autoclave setup for CO_2_ hydrogenation. (b) Catalytic tests in the batch autoclave of Cu(Zn)/Mg_2_(olz) versus CZA at 150–180 °C, 52 bar, CO_2_:H_2_ 1:26, 420 mL autoclave, 22 h in 1 mL dioxane, using 40 mg Cu(Zn)/Mg_2_(olz) or 5 mg CZA.

The Cu(Zn)/Mg_2_(olz) catalyst outperformed the CZA benchmark at 150 °C, achieving higher methanol (MeOH) productivity (3.9 vs. 2.9 mol MeOH per mol Cu + Zn), corresponding to turnover frequencies (TOFs) of 0.18 versus 0.13 h^−1^. At 180 °C, the CZA catalyst showed a slight advantage (4.0 vs. 2.7 mol), and the MOF‐based system demonstrated comparable catalytic activity (TOF = 0.12 h^−1^). Neither the Cu(Zn) species alone nor the Mg_2_(olz) alone showed measurable activity for methanol formation, indicating that only the combined system is active under these conditions. We attribute the observed activity of Cu(Zn)/Mg_2_(olz) to interactions between the MOF support and the dispersed Cu(Zn) phase, while a direct contribution of Mg_2_(olz) to catalytic turnover cannot be established from the present data. It should be noted that Cu(Zn)/Mg_2_(olz) contains a significantly lower Zn content compared to CZA (Table 1), where ZnO stabilizes Cu and contributes to Cu–ZnO interfacial sites. The lower Zn loading in Cu(Zn)/Mg_2_(olz) may therefore limit such interactions and influence the catalytic performance. Consequently, the comparison reflects performance under identical reaction conditions rather than equivalent active phase composition. Although these results do not establish an overall advantage over CZA, they demonstrate the potential of the relatively unexplored Cu(Zn)/Mg_2_(olz) system for lower‐temperature methanol synthesis, with further opportunities for catalyst optimization. Further quantitative comparison with some literature catalysts is provided in the Supporting Information (Table S2).

To further understand the origin of activity of the MOF‐based catalyst in methanol production, we investigated the retained adsorbates, possibly involved in the formation of methanol. ^1^H NMR spectra were collected for the pristine Mg_2_(olz), the fresh Cu(Zn)/Mg_2_(olz), and the Cu(Zn)/Mg_2_(olz) after batch testing at 150 °C (Figure 5). Signals [43] assigned to the Mg_2_(olz) framework appear as broad resonances in the aromatic region (7.0–8.5 ppm, the gray highlight). Residual solvent peaks are visible in the fresh material, including dioxane at ~3.6 ppm (orange highlight) and dimethyl sulfoxide (DMSO) at 2.5 ppm (pink). The fresh Cu(Zn)/Mg_2_(olz) also shows a distinct acetonitrile signal at 2.0 ppm (*), originating from the impregnation step. After the reaction, the spectrum exhibits an additional signal at 3.3 ppm, consistent with methanol (inset) and overlapping with the dioxane region. The peak at 8.3 ppm (•) is assigned to oxygenated species such as formate, while the resonance at 3.7–3.8 ppm indicates methylformate. The presence of methanol and oxygenate signals demonstrates that reaction products are formed within the catalyst structure under reaction conditions, in line with the methanol‐related surface intermediates observed by in situ DRIFTS (vide infra).

**FIGURE 5:**
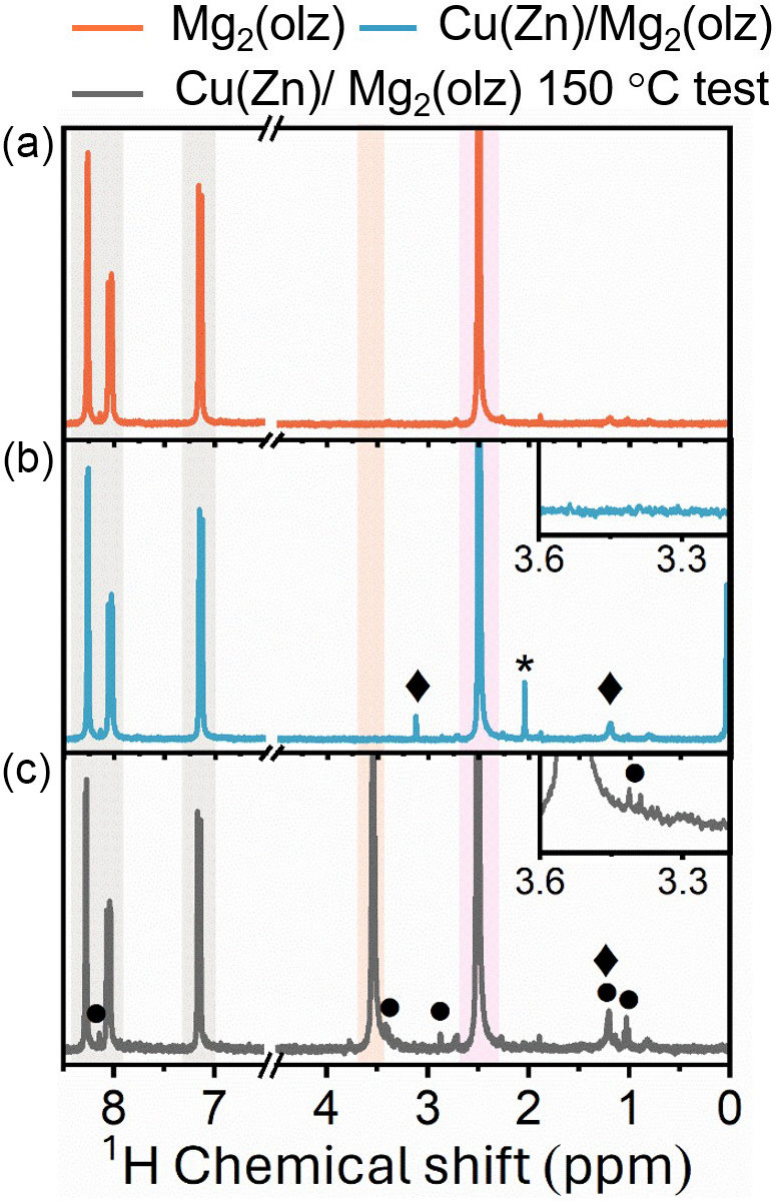
(a) ^1^H NMR spectra of the Mg_2_(olz), (b) Cu(Zn)/Mg_2_(olz), and (c) used Cu(Zn)/Mg_2_(olz) catalyst after test in batch at 150 °C. The signals: Mg_2_(olz) (gray highlight), dioxane (orange), DMSO (pink), possible methanol signal is in insets (b and c); • oxygenates incl. formates and methoxy species, ♦ ‐CH_3_, * acetonitrile from wet impregnation.

Along with this, for the Cu(Zn)/Mg_2_(olz) catalyst, the BET surface area decreases from 1470 m^2^/g before the reaction to 1200 m^2^/g after the batch CO_2_ hydrogenation test at 150 °C (Figure S3). The N_2_ adsorption isotherm retains its characteristic microporous profile, although the lower uptake indicates reduced pore accessibility. In agreement with the postreaction ^1^H NMR spectra (Figure 5c), this reduction may result from partial pore blockage or surface coverage by retained reaction species and residual dioxane. The main Mg_2_(olz) diffraction reflections are also retained, although changes in relative intensities and additional features are observed (Figure S17a). Similar diffraction changes have been linked to solvent occupancy during MOF activation [17, 44, 45, 46], although partial framework degradation or collapse cannot be excluded.

The presence of methanol and oxygenate signals demonstrates that reaction products are formed and retained within the catalyst structure. The dioxane solvent likely facilitates their desorption and removal, shifting the reaction equilibrium by stabilizing methanol and intermediates rather than acting solely as a dispersion medium [47, 48, 49, 50, 51]. These findings show that Mg_2_(olz) retains CO_2_‐derived species within its microporous network but do not establish a direct link between retained species and catalytic turnover. To further understand the performance of the MOF‐based catalyst under reaction‐relevant conditions, transient step‐response experiments were conducted under solvent‐free flow conditions to isolate the gas–solid adsorption behavior from solvent effects.

To monitor the formation and evolution of reaction intermediates over the MOF‐based catalyst, we performed DRIFTS experiments in situ. The experiments included the reaction in the gas phase at 20 bar and 180 °C, followed by the switching cycles between CO_2_ adsorption conditions (CO_2_ + Ar) to reaction conditions (CO_2_ + H_2_ + Ar), then to CO_2_‐free feed (H_2_ + Ar), followed by switching back to reaction feed (CO_2_ + H_2_ + Ar; Figures 6 and S8–S10). To better distinguish the signal of possible reaction intermediates from the MOF signals, we also measured a reference sample with comparable Cu(Zn) loading based on Mg_2_(dobpdc) (MOF‐74‐II, 4,4′‐dioxidobiphenyl‐3,3′‐dicarboxylate linker) [52], which shows comparable properties; further details are provided in the Supporting Information. We measured the same DRIFTS experiment over this sample (Figures S12–S14).

**FIGURE 6:**
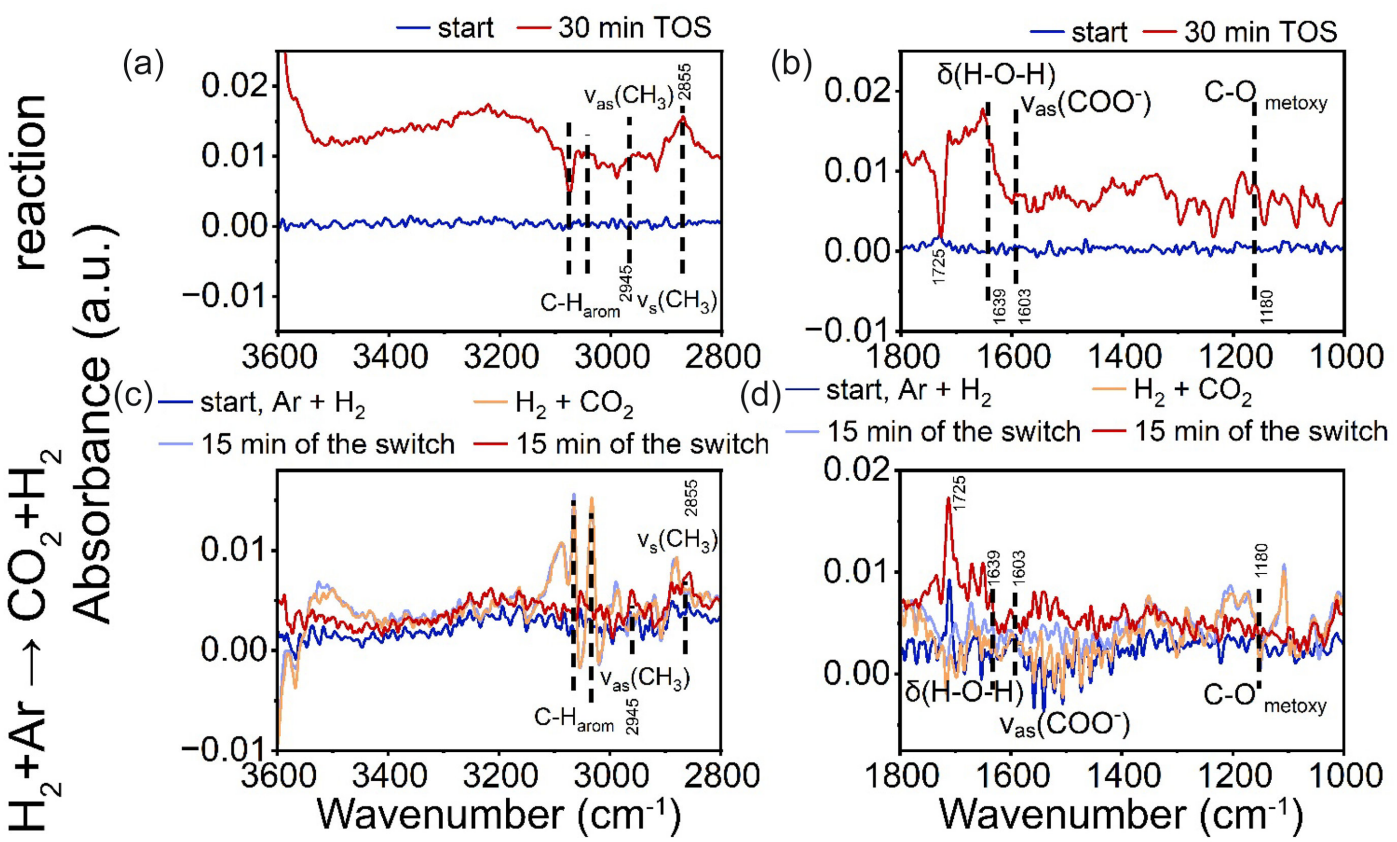
In situ DRIFTS spectra recorded over Cu(Zn)/Mg_2_(olz) under CO_2_ hydrogenation conditions. (a and b). Reaction (CO_2_ + H_2_) and (c and d) transient switching to CO_2_‐free feed (H_2_ + Ar) and back to the reaction feed. Spectra are shown in selected regions: O─H stretching + C─H stretching (a and c, 3600–2800 cm^−1^), and the fingerprint region (b and d, 1800–1000 cm^−1^). Conditions: in situ cell, 20 bar, CO_2_:H_2_ 1:3 (or Ar as a replacement gas), 200 °C pretreatment, 180 °C reaction, 15 mL/min flows.

In situ DRIFTS measurements performed under gas‐phase CO_2_ hydrogenation conditions demonstrate the formation of methanol‐related intermediate species on both Cu(Zn)/Mg_2_(olz) and Cu(Zn)/Mg_2_(dobpdc) catalysts (Figures 6b and S13d). Bands due to the *ν*_as_(COO^−^) mode of formate species develop together with a band at ca. 1180 cm^−1^ that is assigned to C–O of methoxy species [53]. These features evolve together with the pronounced bands in the 3000–2850 cm^−1^ region [54, 55] (Figures 6a and S13c). These changes are consistent with the formation of CH‐containing surface species typically observed during CO_2_ hydrogenation on Cu‐based catalysts [53, 56]. The band at ~1725 cm^−1^ initially develops but becomes strongly masked after 30 min under CO_2_ + H_2_ (Figure 6b); its reversible changes during gas switching (Figures S10b,d and S14b,d) and reappearance upon restoring the reaction feed indicate a transient intermediate that forms and is further consumed under reaction conditions. Importantly, the reference catalyst based on a MOF with a different linker but comparable Cu(Zn) loading shows the same set of spectral features (Figures S13c,d – reaction and S14b,d – switches), thus strengthening the assignment of the weak bands in the original Cu(Zn)/Mg_2_(olz) sample. The presence of the CH‐ stretching region in this reference system supports its attribution to reaction‐related methoxy species rather than to the Mg_2_(olz) MOF framework alone. Gas‐switching experiments further demonstrate the stability of the formed species (Figures 6c,d, S10, and S14). Upon switching from the reaction mixture to CO_2_ + Ar, the intermediates, described by bands in the 1600–1650 cm^−1^ [57, 58] and 1100–1150 cm^−1^ [59] regions, are unaffected (Figures 6d, S10b,d and S14b,d), showing that part of the adsorbed species remains retained on the material on the timescale of the experiment. Overall, the two catalyst formulations follow the same type of surface chemistry, with consistent spectral features observed for the reference catalyst based on an MOF with a different linker but comparable Cu(Zn) loading. This indicates that the observed methoxy intermediates arise from Cu‐based reaction sites rather than from the MOF framework or a different reaction pathway.

While DRIFTS provides insight into the nature and stability of reaction intermediates, it does not directly probe CO_2_ interactions under these conditions. We therefore quantified CO_2_ uptake using transient step‐response experiments in a plug‐flow reactor at 40 bar with a CO_2_:H_2_ ratio of 1:30, representing low CO_2_ concentrations (Figure 7a). The bed was alternately exposed to CO_2_ + H_2_ and N_2_ for 30 min each at set temperatures (30–180 °C), while the outlet composition was monitored by online gas chromatography. The desorption response after switching to N_2_ was integrated over time, and the SiC blank response was subtracted to isolate the contribution of the materials (Figures 7b,c and S15; Table 2). For each material, 4–6 consecutive switching cycles were performed on the same catalyst bed, with asynchronous GC sampling used to reconstruct a single transient response following our previously established methodology [61, 62]. The resulting CO_2_ uptake was calculated by integrating the reconstructed response and is expressed in mmol CO_2_/g_cat_. Details of the method are provided in the Supporting Information. The Cu(Zn)/Mg_2_(olz) catalyst exhibited 3–7 mmol CO_2_/g_cat_ uptake under these conditions, comparable to or exceeding MOF‐74 benchmarks at atmospheric pressure (e.g., Mg‐MOF‐74: 10 mmol CO_2_/g_cat_ at 0 °C, 1 bar [63]; hierarchical Mg‐MOF‐74 monolith: 4 mmol CO_2_/g_cat_ at 25 °C, 1 bar [64]; Ni‐MOF‐74 material: 6 mmol CO_2_/g_cat_ at 25 °C, 1 bar [65]). The elevated pressure compensated for the temperature‐induced decrease in physisorption, sustaining significant uptake even at 150–180 °C. The capacity is attributed to CO_2_ binding at open Mg sites in the MOF nodes [66, 67, 68]. Here, Mg is present as coordinated nodes within the MOF‐74 framework and not as MgO; thus, its role is limited to framework‐defined adsorption sites rather than bulk basic promotion typical for MgO in oxide catalysts [60, 69, 70]. We speculate that additional CO_2_ storage may affect the local CO_2_ concentration near Cu(Zn) sites, but its impact on the catalytic turnover requires further investigation. Moreover, H_2_ may compete with CO_2_ for adsorption at open Mg sites in the MOF under H_2_‐rich, CO_2_‐lean conditions, although this effect is not directly established under the present reaction conditions [18, 71, 72]. CO_2_ adsorption measurements for CZA and pristine UiO‐66, a different MOF topology characterized in our previous work [73], were added for comparison (Table 2). Three independent UiO‐66 samples showed average CO_2_ uptake of 5 ± 0.4, 4 ± 0.7, and 4 ± 0.6 mmol CO_2_/g_cat_ at 30, 150, and 180 °C, respectively. Pristine Mg_2_(olz) retained a higher CO_2_ uptake than UiO‐66 across this temperature range. At reaction‐relevant temperatures (150–180 °C), the adsorption capacity of Cu(Zn)/Mg_2_(olz) is comparable to or slightly lower than CZA, while MOF‐74 olz retains higher uptake. However, for CZA (51 wt% Cu vs. 4 wt% Cu in Cu(Zn)/Mg_2_(olz)), CO_2_ conversion under reaction conditions must be considered; the measured values therefore include contributions from adsorption and catalytic consumption and cannot be interpreted solely as physisorption capacity. At 30 °C, Cu(Zn)/Mg_2_(olz) and Mg_2_(olz) exhibit higher CO_2_ adsorption capacity than CZA, reflecting predominantly physiosorbed CO_2_ under nonreactive conditions, whereas under reaction conditions, the measured values include contributions from chemisorbed and reacting CO_2_ species.

**FIGURE 7:**
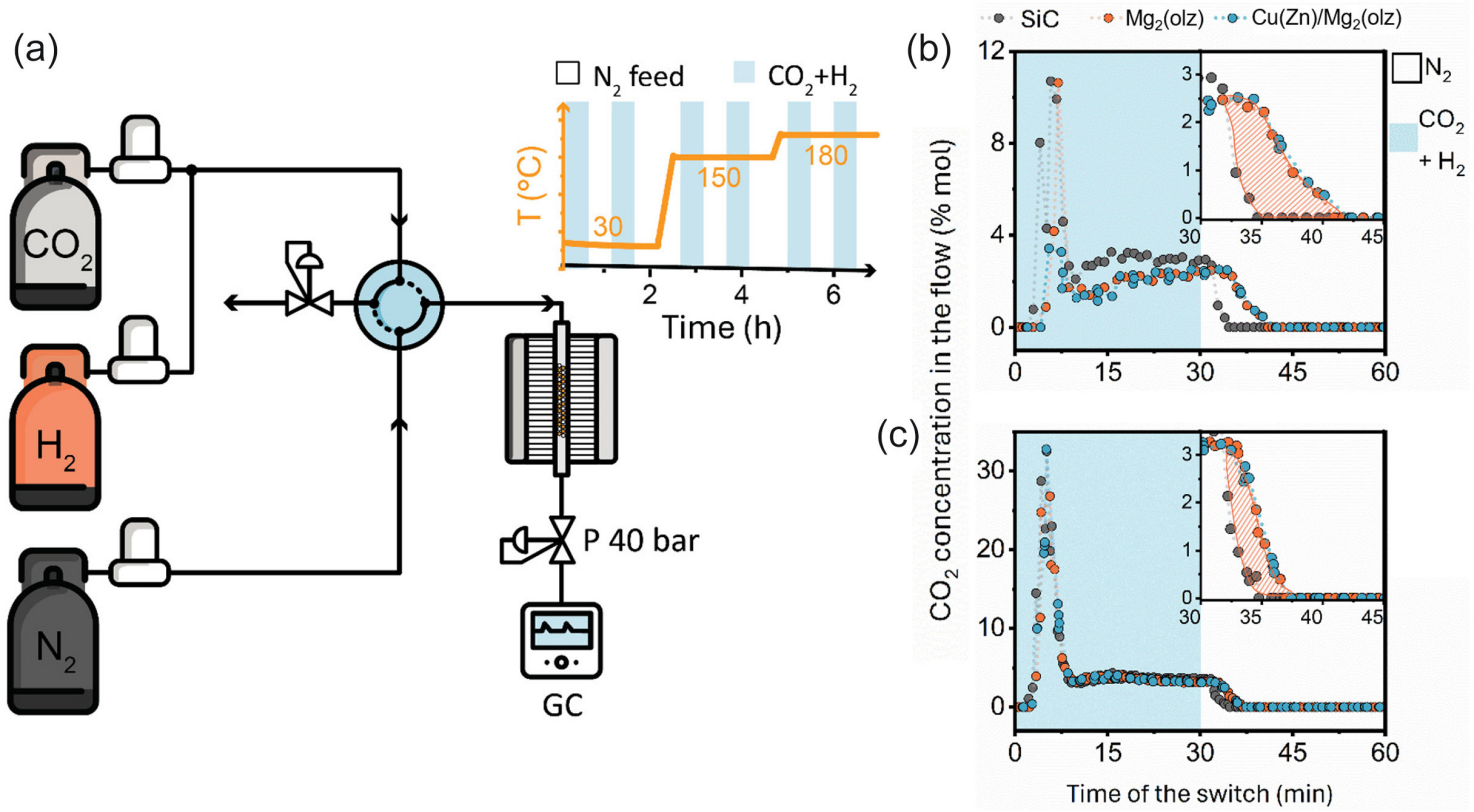
(a) Scheme of the setup for CO_2_ + H_2_ /N_2_ switches and design of the experiment (top right inset); (b and c) the CO_2_ averaged response at 30 and 150 °C. The signal is averaged from 4 to 6 experiments over each material. The integrated area used for the calculation of CO_2_ amount per g of the Mg_2_(olz)‐based material highlighted with orange. Conditions: CO_2_ + H_2_ /N_2_ switching cycles, 30 min in CO_2_ + H_2_ followed by 30 min in N_2_, monitored via online gas chromatography in a plug‐flow reactor at 40 bar. Feed composition CO_2_:H_2_ 1:30, total flow 50 mL/min, temperature range 30–180 °C, catalyst loading 30–40  + 100 mg SiC or 100 mg of SiC only.

**TABLE 2:** Amount of CO_2_ adsorbed during the CO_2_+H_2_ /N_2_ switches for the MOF‐based materials and CZA over different temperatures given in mmol CO_2_/g_cat_.

Sample	30 °C	150 °C	180 °C
Mg_2_(olz)	7	5	5
Cu(Zn)/Mg_2_(olz)	7	4	3
CZA	4	4	3
UiO‐66^a^ [60]	5 ± 0.4	4 ± 0.7	4 ± 0.6

a Values for UiO‐66 represent the mean ± standard deviation of three independent measurements; other values were obtained from reconstructed transient responses.

^1^H NMR spectroscopy of postreaction samples demonstrated the retention of methanol and formate species (Figure S16). However, no methanol was detected online in the gas phase under these conditions over Cu(Zn)/Mg_2_(olz). This can be caused by kinetically limited desorption in the absence of a solvent phase, which may be related to the high surface area of the MOF‐based catalyst (~1500 m^2^/g) and a possible strong binding of oxygenates at open Mg sites of the MOF. These results suggest that under gas‐phase, solvent‐free conditions, product desorption is limited, and the presence of a liquid phase (e.g., dioxane) facilitates the removal of reaction products without evidence for altering the catalytic pathways [49, 74, 75]. While the MOF‐based catalyst stores CO_2_ and stabilizes intermediates, the absence of online methanol detection indicates that reaction conditions require further optimization for effective turnover.

Having assessed the catalytic performance, the composition of the reaction intermediates, and interaction of the materials with CO_2_ under both batch and flow conditions, we next investigated the structural and chemical state of the Cu(Zn) species. Cu and Zn K‐edge XAS were performed, while synchrotron XRD was employed in parallel to monitor the framework structure under in situ reaction conditions. No distinct crystalline Zn‐containing phases were resolved by XRD (Figure S17a). Zn K‐edge XANES showed ZnO‐like features compared with metallic Zn and Cu─Zn alloy references, with no pronounced changes after the reaction [73]. However, the low Zn loading (0.2 wt.%) prevented reliable quantitative XANES and EXAFS analyses, leaving minor metallic Zn or Cu–Zn alloy contributions unresolved. Consequently, the present data do not establish whether Zn acts as a structural or electronic promoter or remains a spectator during the catalysis. Cu K‐edge XAS of the fresh and used Cu(Zn)/Mg_2_(olz) catalyst was used to investigate the oxidation state of copper before and after batch catalysis (Figures 8 and S18–S20). The XANES spectra of the fresh material showed features resembling Cu_2_O, with a pronounced white line and edge position consistent with a predominantly Cu(I) phase. Upon reaction, the edge shifted to lower energy and the white line intensity decreased, evidencing partial reduction to metallic Cu^0^ (~90% Cu^0^ ~10% Cu_2_O from the linear combination fitting (LCF); the LCF fit is provided in Figure S18b). In the corresponding Fourier‐transformed EXAFS, the fresh catalyst exhibited a dominant Cu─O peak (~1.5 Å, phase‐uncorrected), while after reaction, a strong Cu–Cu peak (~2.2–2.5 Å) emerged, consistent with metallic copper. Quantitative EXAFS fitting (Figures 8c,d and S20; Table 3) shows that the fresh Cu(Zn)/Mg_2_(olz) is described by a Cu─O shell at ~1.90 Å with a low effective coordination number (*N*_av_ ≈ 1.4), indicating a locally under‐coordinated Cu(I) environment. A weak Cu─Cu contribution at ~3.0 Å a limited medium‐range order in this state. After the reaction, the EXAFS is well reproduced by a metallic Cu model with a Cu─Cu distance of 2.54 Å and a high coordination number (*N*_av_ ≈ 9.6), consistent with the formation of metallic Cu domains. The reduced coordination number compared to bulk Cu reflects finite size and/or structural disorder, in agreement with TEM observations of Cu(Zn) domains (Figures 3 and S5), and coincides with the reaction intermediates observed by in situ DRIFTS and post‐reaction ^1^H NMR, without implying direct involvement of the MOF framework in the catalytic step.

**FIGURE 8:**
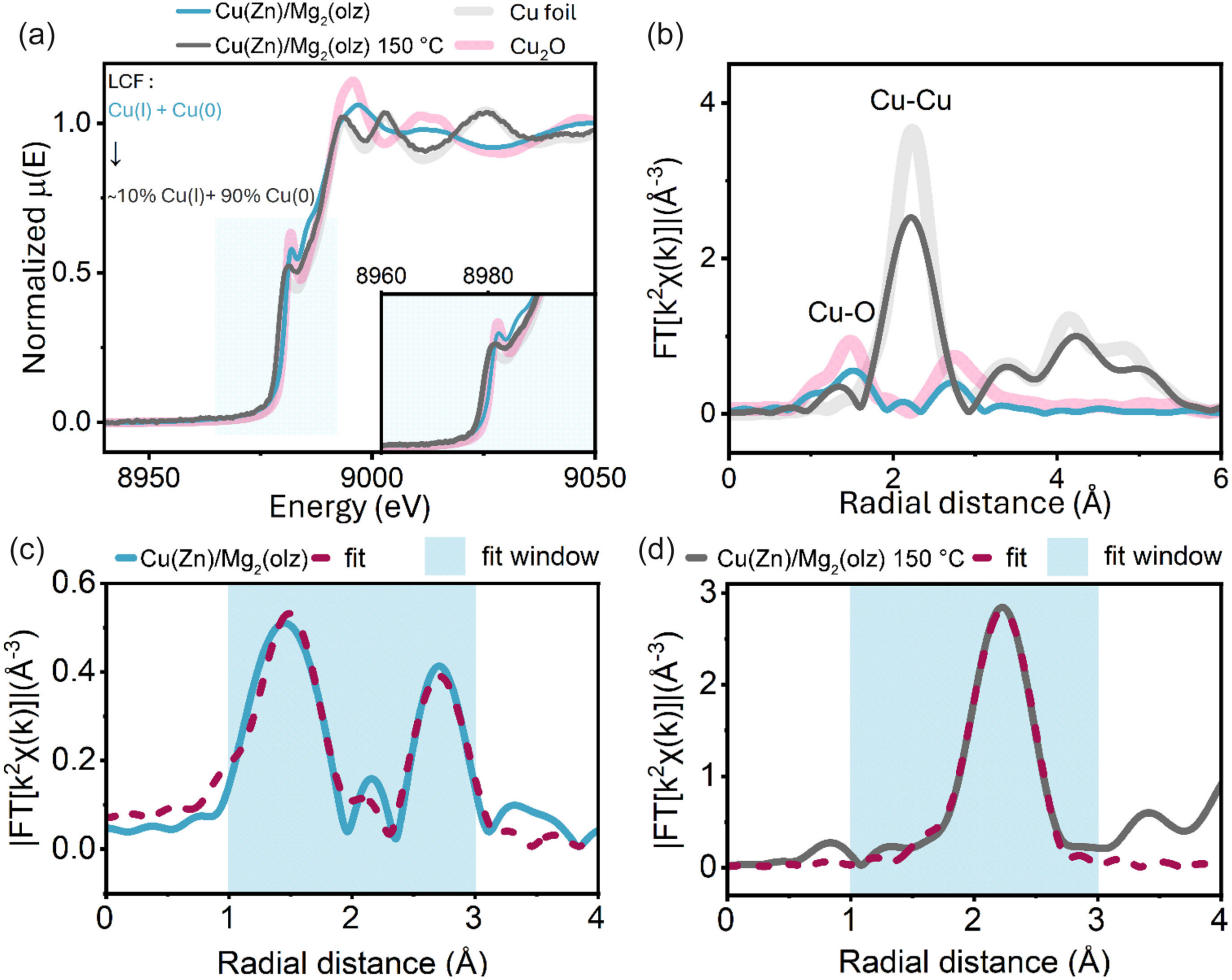
(a) Cu K edge XANES and (b) phase uncorrected |FT‐EXAFS| spectra of the fresh and used catalyst. Cu foil and Cu_2_O used as references. (c and d) **|**FT| of averaged *k* ^2^‐weighted EXAFS spectra and their fit of the Cu species in Cu(Zn)/Mg_2_(olz) and Cu(Zn)/Mg_2_(olz) used at 150 °C in batch samples. The inset in (a) shows an enlarged view of the XANES preedge region. Experimental and best fit curves of imaginary components and EXAFS spectra are reported in Figure S19.

**TABLE 3:** Fit results of the averaged spectra of the Cu species in Cu(Zn)/Mg_2_(olz) and Cu(Zn)/Mg_2_(olz) used in batch at 150 °C.

Sample	Path	*N*	*S*_0_^2^	*N*_av_	σ^2^, Å^2^	*E*_0_, eV	*R*, Å	*R*‐factor
Cu foil	Cu─Cu	12	0.90 ± 0.05 (fitted)	12	0.0088 ± 0.0005	4.129	2.54 ± 0.003	0.003
Cu(Zn)/Mg_2_(olz)	Cu─O	2	0.9 (fixed)	1.4	0.0048 ± 0.0008	8	1.90 ± 0.03	0.03
	Cu─Cu	12	0.9 (fixed)	2.4	0.0080 ± 0.007		3.00 ± 0.003	
Cu(Zn)/Mg_2_(olz) used 150 °C	Cu─Cu	12	0.9 (fixed)	9.6	0.0087 ± 0.0007	4	2.54 ± 0.005	0.006

In situ Cu K‐edge XANES under CO_2_ hydrogenation conditions (Figures 9a,b and S21) corroborated this redox evolution. Spectra, referenced using Cu, Cu_2_O, and CuO standards, showed a gradual shift from the mix of Cu(II) + Cu(I)‐like to metallic Cu features upon activation in a H_2_‐rich mixture. Linear combination fitting of the time‐resolved data indicated rapid reduction during the initial activation stage, with the metallic fraction remaining stable during subsequent CO_2_ hydrogenation at 180 °C and 10 bar of total pressure. Switching between the CO_2_ + H_2_ feed and He + H_2_ after 2 h of reaction did not alter the Cu speciation [76], indicating that a predominantly reduced Cu phase is maintained under reaction conditions (Figure 9b). Notably, the difference in the initial oxidation state Cu(I) in Cu(Zn)/Mg_2_(olz) before the batch test in Figure 8 and Cu(II) in the in situ experiment in Figure 9b likely reflects the inhomogeneous distribution of the Cu(Zn) species, as also suggested by the XRD patterns in Figure S4a, showing both oxide and metallic Cu phases. Alternatively, this difference may reflect sample heterogeneity and partial reoxidation of Cu(Zn) species, as the catalyst used for the in situ experiment was prepared and measured considerably later than that used for the other measurements. Nevertheless, in both cases, the catalyst evolved under reaction conditions to a stable mixture of ~90% metallic Cu and ~10% Cu(I). The rapid reduction during activation is consistent with the Cu reduction event observed by H_2_‐TPR at approx. 175 °C. Simultaneously collected in situ synchrotron XRD patterns showed that the Cu(Zn)/Mg_2_(olz) framework maintained its crystallinity throughout activation and CO_2_ hydrogenation (Figure 9c). The diffraction peaks remained sharp and unchanged up to 200 °C, with no signs of amorphization or collapse, consistent with the TGA‐MS results, indicating that framework decomposition starts only above the catalytic temperature range.

**FIGURE 9:**
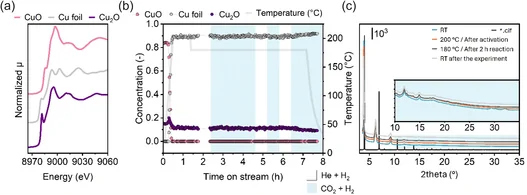
(a) XANES Cu K edge spectra of the Cu, Cu_2_O, CuO references for the linear combination fitting; (b) LCF analysis of XANES Cu K edge collected over Cu(Zn)/Mg_2_(olz) in gas‐phase CO_2_ hydrogenations conditions. Conditions: capillary cell, 10 bar, CO_2_:H_2_ or He:H_2_ 1:3, 180 °C reaction, 30 mL/min. (c) XRD patterns collected simultaneously in the same experiment. XRD data collected at 10 keV and transformed to Cu Kα wavelength. Mg_2_(olz) with olsalazine linker referenced in gray. The inset in (c) shows an enlarged view of the XRD patterns at higher angles.

Experimental data indicate that CO_2_ remains within the MOF pores during the reaction and can possibly interact with the Cu^0^–ZnO species formed in situ. These Cu(Zn) species are associated with the formation of methanol intermediates. The framework stays structurally ordered under the applied conditions, and the Cu(Zn) domains show no significant growth under the measured hydrogenation conditions. While the present results show the coexistence of CO_2_ adsorption and catalytic activity in the MOF‐based catalyst, the direct role of adsorbed CO_2_ in the catalytic cycle requires further investigation. Future work should focus on decoupling adsorption and reaction under both gas‐ and liquid‐phase conditions [49, 74], probing the role of Mg^2+^ sites (site blocking [77] or linker modification [20, 21]), and establishing kinetic correlations between CO_2_ uptake and methanol formation. The difference between liquid‐phase batch and gas‐phase flow conditions suggests that solvent‐mediated effects on product desorption and intermediate stabilization influence the observed catalytic behavior. Overall, MOF‐based catalysts represent an emerging class of materials with potential coexisting adsorption–conversion functionality, for which this study provides an initial experimental basis.

## 4 Conclusion

We tested a Cu–Zn‐modified Mg_2_(olz) catalyst for CO_2_ hydrogenation to methanol under high pressure and low CO_2_ concentrations relevant to dilute industrial CO_2_ streams. Compared to the commercial Cu/ZnO/Al_2_O_3_ catalyst, the MOF‐based catalytic material showed 34% higher activity at 150 °C in a batch test. Alternatively, it showed the same activity as the benchmark material but at 30 °C lower temperature, setting a significant energy‐saving factor. In situ DRIFTS and ^1^H postreaction NMR spectroscopy identified methanol‐related surface intermediates over the MOF‐based catalysts. Step‐response experiments under continuous flow showed reversible CO_2_ uptake of 3–7 mmol CO_2_/g_cat_ between 30 and 180 °C, in a range of temperatures relevant for adsorption and conversion of CO_2_. XAS analysis revealed partial reduction of the introduced copper species to metallic Cu after the reaction, while in situ XRD demonstrated that the framework remained stable up to 200 °C.

By combining catalytic testing, adsorption measurements, and spectroscopic probes, this work defines how an MOF‐based catalyst operates under low CO_2_ partial pressures and elevated total pressures, distinguishing CO_2_ adsorption within the framework from catalytic transformation on Cu(Zn) sites. The study shows that CO_2_ adsorption and hydrogenation coexist within an MOF‐based system, providing a basis for further quantitative investigation through systematic material modifications. It sets out methods to evaluate active metal speciation and stability of the metal‐framework‐based materials under reaction conditions. These results offer the community practical insight into how MOFs can be developed and tested for combined adsorption–conversion applications.

## Funding

This study was supported by Schweizerischer Nationalfonds zur Förderung der Wissenschaftlichen Forschung (204284).

## Conflicts of Interest

The authors declare no conflicts of interest.

## Supporting information

The following files are available free of charge: Detailed characterization and synthesis procedures for Mg_2_(olz), Mg_2_(dobpdc), Cu(Zn) species, and Cu(Zn)/Mg_2_(olz) catalyst preparation; synthesis schemes; ATR‐IR spectra; N_2_ physisorption isotherms and textural properties; H_2_ temperature‐programmed reduction of Cu(Zn) species; STEM‐EDX and TEM characterization; ex situ XRD patterns; photograph of the catalyst suspension in dioxane; catalytic benchmarking table; DRIFTS experimental setup, spectra, gas‐switching experiments, and comparison with Cu(Zn)/Mg_2_(dobpdc); methodology and quantification of transient CO_2_ step‐response adsorption experiments; ^1^H NMR spectra after adsorption tests; Zn K‐edge XANES; Cu K‐edge XAS spectroscopy including XANES linear‐combination fitting, EXAFS fitting parameters and models, reference measurements, and supplementary references (PDF).

## Data Availability

The data that supports the findings of this study are available in the Supporting Information of this article.
