# Supplementary material for: Structure–Performance Relationships in Metal–Organic Framework Mg2(olz) Catalysts for CO2 Adsorption and Hydrogenation to Methanol
Source: ChemSusChem. 2026 Nov 5;19(21):e71202. doi: 10.1002/cssc.71202 (PMC13738046; doi:10.1002/cssc.71202)
Supplement: Supplementary file 1 [file CSSC-19-e71202-s001.pdf]

**Supporting information**

to

# Structure-Performance Relationships in Metal-Organic Framework $Mg_2$(olz) Catalysts for $CO_2$ Adsorption and Hydrogenation to Methanol

*Anna Liutkova[a*], Fabio André Peixoto Esteves[a,b], Anastasia Molokova[c], Marianela López Romero[a,c], Davide Salusso[c], Andrea Testino[a], Emiliya Poghosyan[d], Marco Ranocchiari[a*]*

[a] Center for Energy and Environmental Sciences, PSI, Forschungsstrasse 111, 5232 Villigen Paul Scherrer Institute PSI, Switzerland

[b] Department of Chemistry and Applied Biosciences, ETH Zürich, Vladimir Prelog Weg 1-5, 8093 Zurich, Switzerland

[c] ESRF, The European Synchrotron, 71 Avenue des Martyrs, 38000 Grenoble, France

[d] Center for Life Sciences, Paul Scherrer Institute PSI, Forschungsstrasse 111, 5232 Villigen PSI, Switzerland

Corresponding authors:

Anna Liutkova
Center for Energy and Environmental Sciences
Paul Scherrer Institute PSI
Forschungsstrasse 111
5232 Villigen PSI, Switzerland
E-mail: anna.liutkova@psi.ch

Marco Ranocchiari
Center for Energy and Environmental Sciences
Paul Scherrer Institute PSI
Forschungsstrasse 111
5232 Villigen PSI, Switzerland
E-mail: marco.ranocchiari@psi.ch

## Contents

1. Materials and methods ........ 3

2. Synthesis of the $Mg_2$(olz) MOF ........ 10

3. ATR-IR ........ 11

4. $N_2$ physisorption ........ 12

5. Synthesis of the Cu(Zn) species and their incorporation into the $Mg_2$(olz) MOF ........ 13

6. Catalytic testing in batch, liquid phase ........ 16

7. In situ DRIFTS under high-pressure $CO_2$ hydrogenation ........ 17

7.1 Cu(Zn)/$Mg_2$(dobpdc) material for the DRIFTS comparison ........ 20

8. Quantification of $CO_2$ adsorption from the step-response experiments ........ 26

9. $^1$H NMR spectra of the retained species after the step-response adsorption experiments ........ 28

10. State of Zinc in Cu(Zn)/$Mg_2$(olz) catalyst ........ 29

11. XAS results - state of Copper in Cu(Zn)/$Mg_2$(olz) catalyst ........ 30

References ........ 32

## 1. Materials and methods

$^1$H NMR spectroscopy: $^1$H spectra of the dissolved samples were recorded on a Magritek Spinsolve 60 MHz spectrometer and a Bruker Avance III 300 MHz spectrometer at room temperature in DMSO-$d_6$+DCl. Spectra were referenced to the signals of DMSO-$d_6$ ($\delta$ = 2.50 ppm).

ATR-IR spectra were recorded on a Vertex 70 (Bruker) covering a range of 400-4000 $cm^{-1}$ with a resolution of 4 $cm^{-1}$ and 32 scans, room temperature, air-exposed.

Ex situ XRD measurements were performed at the ID15A, ESRF synchrotron (Grenoble, France)[1],[2]. The photon wavelength was 0.01937 nm (64 keV). The samples were measured using glass capillaries sealed air-tight in the glovebox.

For TEM sample preparation, a lacey carbon grid (Plano AG, product number S166-3) was used. The sample was first crushed using a mortar inside a glovebox, then dispersed in ethanol and sonicated for a few minutes. Approximately 4 µL of the resulting suspension was deposited onto the grid and allowed to dry on filter paper. TEM and STEM-EDX images were obtained using a probe-corrected JEOL JEM-ARM200F electron microscope operating at 200 kV. To minimize beam-induced damage to the MOF, TEM imaging was performed in low-dose mode. For STEM-EDX measurements, a cold field emission gun (cFEG) was operated at a reduced emission current of 5 µA, resulting in probe currents of approximately 4-10 pA.

TGA-MS was performed using a Mettler Toledo TGA/DSC 1 instrument. An amount of the $Mg_2$(olz) MOF (≈ 5 mg) was placed in an alumina crucible and then heated up to 1000 °C with a ramp rate of 5 °C/min in artificial air flow or in 5% $H_2$ in Ar at 20 mL/min. The TGA chamber outlet was connected to a mass spectrometer (MS, Pfeiffer Thermostar).

Chemical composition was measured by ICP-OES using an Agilent ICP-OES 5110 spectrometer. To dissolve the samples, we used 5-20 mg of the sample, 1 mL of $H_2SO_4$ (>95%, Fisher Scientific AG). Then the samples were sonicated and/or microwaved, diluted 10 times with milliQ water, and transferred to tubes for the measurement.

$N_2$ physisorption measurements: the materials were activated overnight at 0.20 mbar and 200–250 °C, using a Micromeritics VacPrep 061 sample preparation device. The weight loss due to activation was considered for the calculation of molar amounts of materials, and the $N_2$ physisorption experiments were conducted on a Micromeritics 3Flex instrument.

Hydrogen chemisorption: to observe the reduction of the Cu(Zn) species, we performed $H_2$ temperature-programmed reduction in $H_2$ ($H_2$-TPR). The measurement was performed using the Autochem instrument (Micromeritics). About 50 mg of a material was first heated up to 150 °C in Ar to remove the possible moisture, then cooled down and subjected to 5 vol.% $H_2$ by heating with a 10 °C/min ramp to 400 °C. The response was recorded using the TCD detector.

The catalytic performance in the $CO_2$ hydrogenation reaction was evaluated in a batch system. In an air-free atmosphere, 40 mg of the $Mg_2$(olz)-based catalyst (or 5 mg of CZA, corresponding to a comparable amount of (Cu+Zn)) was suspended in 1 mL dry dioxane (99.5+%, Fisher Scientific AG) in a 1 mL glass vial. The vial was then transferred to a 420 mL Parr autoclave equipped with a pressure transducer. Prior to the experiment, the autoclave with the catalyst inside was purged three times with 2 bars of $N_2$ and subsequently pressurized with 2 bar of $CO_2$. Then, 50 bar of $H_2$ were added and after that, the system was heated up to reaction temperature using a heating mantle. The reaction time was 22 h, after that, the system was first cooled down to room temperature, then slowly depressurized and the liquid products were collected. The collected sample was dissolved in 2 mL of dioxane and analyzed using the gas chromatograph (GC) Agilent

6890N Network GC system equipped with a DB-WAXetr column (30 m × 0.25 mm × 0.25 μm) and a thermal conductivity detector (TCD). Helium was used as the carrier gas at a constant flow rate of 2 mL/min. Samples (1 μL) were injected in split mode (20:1 split ratio). The oven temperature was ramped from 70 °C to 160 °C at 20 °C/min and held at 160 °C for 1.5 min. Methanol yields were quantified using an external calibration curve, with reported values representing the average of three injections. The catalytic tests were performed in duplicate. The amount of methanol produced was determined from the calibrated GC peak area and the final liquid volume:

$$n(MeOH) = [A(MeOH)/RF] \times V$$

where A(MeOH) is the integrated methanol peak area, RF is the calibration response factor, and V is the sample volume.

Methanol turnover number (TON) and (Cu+Zn)-normalized space-time yield (STY) were calculated as follows:

$$TON = n(MeOH)/n(Cu+Zn)$$

$$STY(Cu+Zn) = n(MeOH) / [m(Cu+Zn) \times t]$$

where t = 22 h and m(Cu+Zn) is the combined mass of Cu and Zn in the catalyst. TON is expressed in mol MeOH per mol Cu+Zn, apparent TOF in $h^{-1}$, and STY in mmol MeOH / g (Cu+Zn) × h. Both TON and apparent TOF are normalized to the total Cu+Zn amount rather than to the number of accessible catalytic sites. Therefore, the reported TOF represents an average metal-normalized rate over the batch reaction, not an intrinsic site-specific turnover frequency.

The $CO_2$ adsorption experiments in the gas-phase were carried out using a fixed-bed reactor with a feed of $CO_2$ and $H_2$ or $N_2$. In a typical experiment, a stainless-steel reactor with I.D. of 2.5 mm was charged with 30-40 mg of $Mg_2$(olz)-based material or CZA + 100 mg of SiC or 100 mg

of SiC. The reactor was sealed with metal frits with a pore size of 0.5 μm. The catalyst was activated in an $H_2$-containing atmosphere (10 vol.% $H_2$ in $N_2$) at 200 °C (heating rate 10 °C /min) and 5 bar for 4 h to remove possible organic contaminants and adsorbates from air after the transfer from the glovebox, and to reduce the introduced Cu(Zn) species. After the activation, the temperature was lowered to 30 °C in $N_2$ and the system was pressurized to 40 bar. The $CO_2$ capacity of $Mg_2$(olz)-based materials and CZA under dynamic conditions was evaluated with a $CO_2$:$H_2$ feed ratio of 1:30, total flow rate of 50 mL/min, and temperature ramps in a range 30 – 150 – 180 °C with periodic 30 min switches[3] between $N_2$ and $H_2$ + $CO_2$.

Online analysis of the $CO_2$ adsorption experiments was performed by a gas chromatograph (GC, CompactGC 4.0, Global Analyzer Solutions) with the heated gas transfer lines. The GC had four pre-columns, four columns, three detectors and a Methanizer device. A thermal conductivity detector (TCD) coupled with an RT-Q-Bond pre-column (3 m; i.d. 0.32 mm; film thickness 10 μm) and a Molsieve 5A FS column (15 m; i.d. 0.32 mm; film thickness 30 μm) was used for the analysis of the permanent gases including $H_2$. $CO_2$, $N_2$ were analyzed by another TCD coupled with an RT-Q-Bond pre-column (3 m; i.d. 0.32 mm; film thickness 10 μm) and an RT-Q-Bond column (12 m; i.d. 0.32 mm; film thickness 10 μm). Possible oxygenates were traced using an MXT-5 column (12 m; i.d. 0.28 mm; film thickness 1 μm) coupled with MXT-5 pre-column (3 m; i.d. 0.28 mm; film thickness 1 μm) and analyzed with a flame ionization detector (FID). A separate channel connected to a Methanizer device and later to the FID detector was used for possible low CO concentrations. The channel is equipped with a Carboxen 1010 pre-column (3 m; i.d. 0.32 mm) and Carboxen 1010 column (12 m; i.d. 0.32 mm).

In situ DRIFTS experiments were performed using a high-pressure reaction cell coupled to an FTIR spectrometer, equipped with a liquid-$N_2$-cooled MCT detector. We followed the setup

described previously for in situ studies of $CO_2$ hydrogenation catalysts (Fig. S7).[4] The catalyst sample was loaded into the DRIFTS cell and purged with Ar prior to activation. The catalyst was pretreated under a $H_2$ + Ar mixture (5 bar) while heating to 200 °C. The sample was held under these conditions for 30 min to activate the catalyst. After pretreatment, the cell was pressurized to 20 bar and the temperature was set to 180 °C for the reaction. After 30 min of reaction, gas-switching experiments were performed to probe the interaction of $CO_2$ and $H_2$ with the catalyst surface under reaction conditions. Overall, after the pretreatment, the catalyst was sequentially exposed to the following gas mixtures: Ar + $CO_2$ + $H_2$ (reaction mixture) for 30 min; Ar + $CO_2$ ($CO_2$ adsorption conditions, no $H_2$) for 15 min; Ar + $CO_2$ + $H_2$ (reaction mixture) for 15 min; Ar + $H_2$ (hydrogen atmosphere) for 15 min, and the last one, Ar + $CO_2$ + $H_2$ (reaction mixture) for 15 min. The total flow rate was maintained at approximately 15 mL/min, with gas flows adjusted to obtain representative reaction compositions ($H_2 \approx 12$ mL/min, $CO_2 \approx 3$ mL/min, with Ar used as balance gas when required). Spectra were recorded continuously during the gas-switching experiments, with a spectral resolution of 4 $cm^{-1}$, averaging 40 scans per spectrum, and a scanner velocity of 10 kHz. A new background was taken at the start of pretreatment, start of reaction, and before Ar + $CO_2$ switch.

The ex situ and in situ characterization of the retained Cu(Zn) species, their local environment and the chemical state was carried out using XAS combined with XRD for the in situ measurements at BM23, ESRF[5]. The storage ring operated in 16-bunch top-up mode at 80 mA maximum current. Measurements were performed in transmission at Cu K-edge and Zn K-edge, using a double-crystal Si(111) monochromator moving in continuous mode followed by Si-coated double reflection mirrors for harmonic rejections. The acquisition time for one XAS spectrum was 90 seconds, with an energy range of 8800 – 10900 eV. Ionization chambers were used to detect

incident and transmitted photons. I0 chamber was filled with 1.57 bar $N_2$, I1, and I2 with 0.33 bar Ar, all three topped up with He at a total pressure of 2 bar. The spectra of the Cu reference foil placed between I1 and I2 were measured together with the sample and then used for energy alignment. Spectra energy alignment, background subtraction, edge jump normalization and EXAFS extraction were performed with Larch-based python scripts.[6] EXAFS Fourier Transform was performed in the 3 – 12 $Å^{-1}$ k-range with Hanning window. XANES Linear combination fit (LCF) was performed in the $E_0 \pm 50$ eV range using metallic Cu, $Cu_2O$ and CuO references. The weight of each component was forced to be positive while the components sum was forced to 1.

The in situ catalytic hydrogenation of $CO_2$ combined with XAS + XRD was carried out using the gas delivery and reaction system available in-house. During the in situ experiment, the catalyst was activated in an $H_2$-containing atmosphere while heating to 200 °C, followed by $CO_2$ hydrogenation at 180 °C and 10 bar total pressure using a $CO_2$:$H_2$ ratio of 1:3 and a total flow rate of 30 mL/min. After approximately two hours under reaction conditions, the feed was switched from $CO_2$+ $H_2$ to He + $H_2$ while maintaining the same temperature and pressure. The setup allows control over reactant composition, flow rates, pressure, and temperature under reaction conditions compatible with in situ XAS. The gas feed lines were connected to high-purity $CO_2$, $H_2$, and He cylinders, each regulated by individual calibrated Bronkhorst mass flow controllers. Helium was used as the inert carrier gas. The individually controlled gas streams from $CO_2$, $H_2$, and He sources were combined downstream of the mass flow controllers into a common mixing line. This line was directed to a four-way switching valve, which allowed the gas mixture to be routed either toward the reactor cell or diverted to exhaust. The reactor consisted of a quartz capillary (1.5 mm O.D.), which was packed with quartz wool and the catalyst sample. This capillary, enclosed in the sample holder, was aligned in the X-ray beam path. The reactor was heated with a gas blower; the outlet

was connected to a Bronkhorst back-pressure regulator, and the gas composition was continuously monitored with a mass spectrometer during the XAS + XRD in situ experiment. XRD was measured at various stages of the experiment to observe the $Mg_2$(olz) MOF stability. XRD was measured at 10 keV with a Pilatus 1M detector, which operates in single photon counting mode.

## 2. Synthesis of the $Mg_2$(olz) MOF

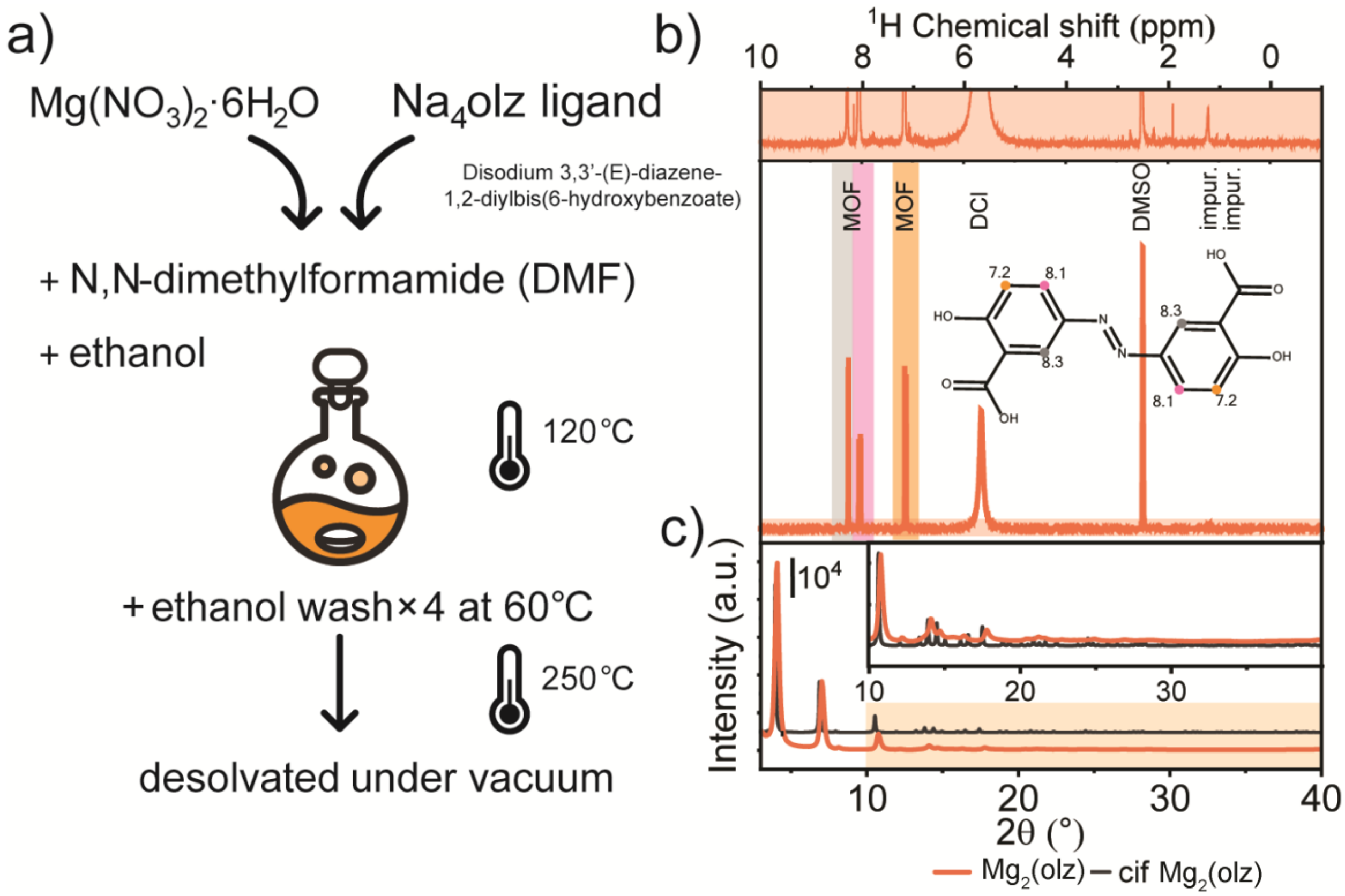


**Figure S1.** a) Scheme of the synthesis of the $Mg_2$(olz) MOF; b) $^1$H NMR spectra of the synthesized $Mg_2$(olz) with the signals from the linker highlighted with grey, pink, orange. The insert corresponds to the enlarged region to observe the remaining solvents; c) XRD of the MOF compared with the simulated pattern calculated from a modified CIF[7] containing only the $Mg_2$(olz) framework after removal of pore guests. The insert corresponds to the enlarged region with higher angles. The XRD data were collected at 64 keV and transformed to Cu Kα wavelength.

**Procedure:** $Mg(NO_3)_2{\cdot}6H_2O$ (4.53 g, 17.67 mmol, 2.1 equiv.; Sigma-Aldrich Chemie GmbH, ≥99.0%) was dissolved in ethanol (224 mL; VWR International GmbH, analytical grade), while $Na_2$(olz) (2.9996 g, 8.40 mmol, 1.0 equiv.; Chemie Brunschwig AG, ≥98%) was dissolved in N,N-dimethylformamide (DMF; 396 mL; Chemie Brunschwig AG, HPLC grade). The ligand solution

was sonicated for 15 min to aid dissolution. The two solutions were combined in a sealed borosilicate reactor equipped with a pressure-release line (bubbler), resulting in a total reaction volume of 620 mL and a concentration of ~0.014 M. The reaction mixture was heated from 30 °C to 120 °C at 10 °C/min and maintained at 120 °C for 23 h under static conditions. After cooling, the resulting solid was isolated by filtration. The product was washed with DMF at 80 °C and ethanol at 60 °C. Solvent exchange was performed in ethanol (800 mL per cycle) at 60 °C for multiple cycles (18 - 70 h per cycle) until removal of residual DMF and ethanol was confirmed by $^1$H NMR. Activation was carried out under dynamic vacuum at 250 °C (~70 h). The fully activated material was obtained as a green powder.

## 3. ATR-IR

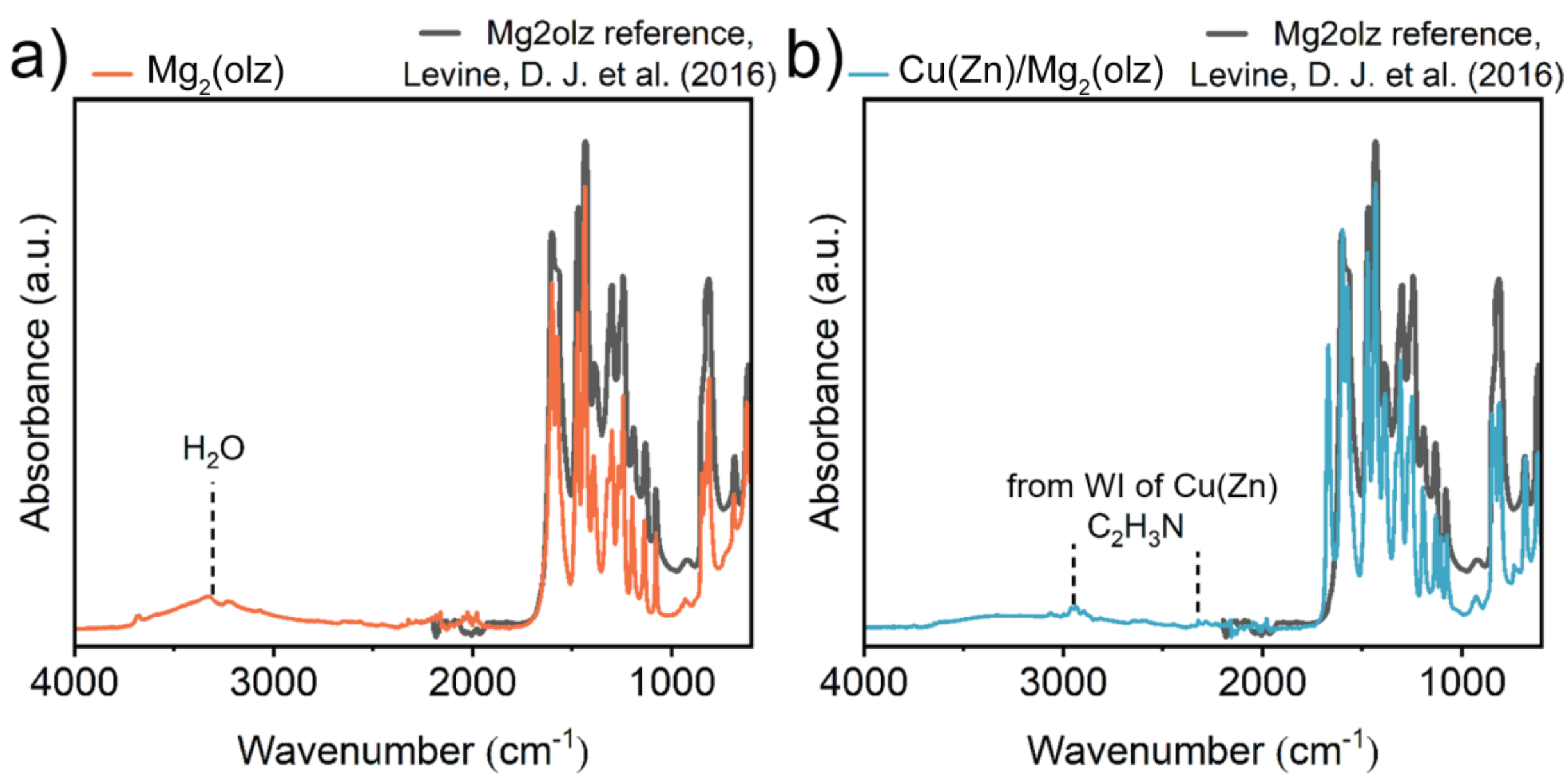


**Figure S2.** ATR-IR of the $Mg_2$(olz) MOF (a) and Cu(Zn)/$Mg_2$(olz) (b) materials compared to a reference material.[8] WI – wet impregnation. The wide IR band at ~3400 cm$^{-1}$ in the pristine $Mg_2$(olz) MOF is attributed to rapid $H_2O$ adsorption from air at open metal sites, whereas its absence in Cu(Zn)/$Mg_2$(olz) indicates that these sites can be partially occupied by residual

acetonitrile from impregnation, limiting water uptake.

## 4. $N_2$ physisorption

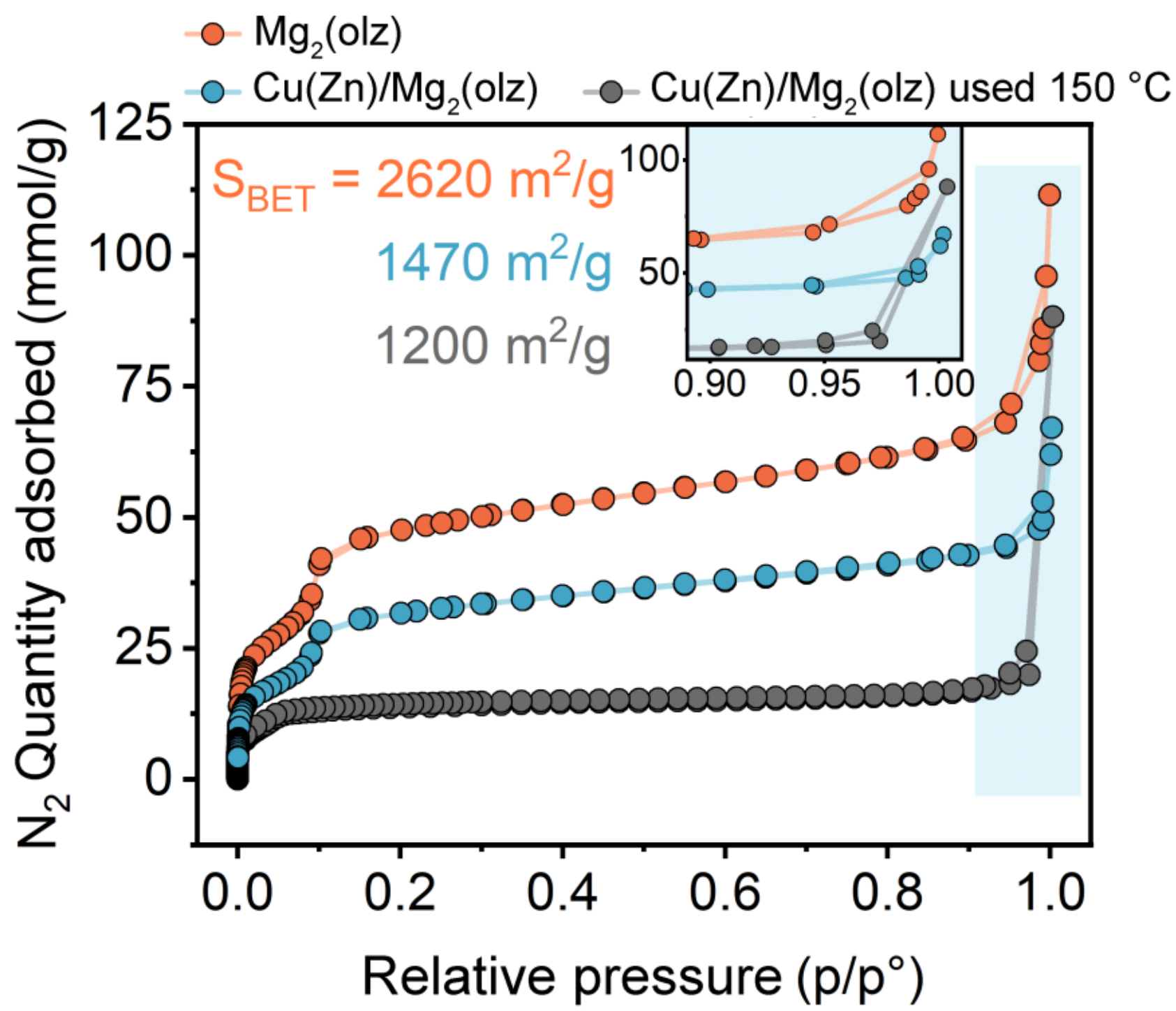


**Figure S3.** $N_2$ adsorption isotherms of the $Mg_2$(olz) (orange), Cu(Zn)/$Mg_2$(olz) catalyst, and used Cu(Zn)/$Mg_2$(olz) catalyst after a test in batch at 150 °C. The inset corresponds to the mesoporous region ($p/p^0 \approx 0.9$-1.0), demonstrating partial retention of mesoporosity after catalysis.

**Table S1.** $N_2$ physisorption results of the $Mg_2$(olz)-based materials.

| **Sample** | $S_{BET}$, ($m^2$/g) | $S_{micro}$, ($m^2$/g) | $V_{total}$ ($cm^3$/g) | $V_{micro}$ ($cm^3$/g) |
|---|---|---|---|---|
| $Mg_2$(olz) | 2620 | 2370 | 1.9 | 1.4 |
| Cu(Zn)/$Mg_2$(olz) | 1470 | 1160 | 1.3 | 0.6 |
| Cu(Zn)/$Mg_2$(olz) after a test in batch at 150 °C | 1200 | 1010 | 0.6 | 0.3 |

5. **Synthesis of the Cu(Zn) species and their incorporation into the $Mg_2$(olz) MOF**

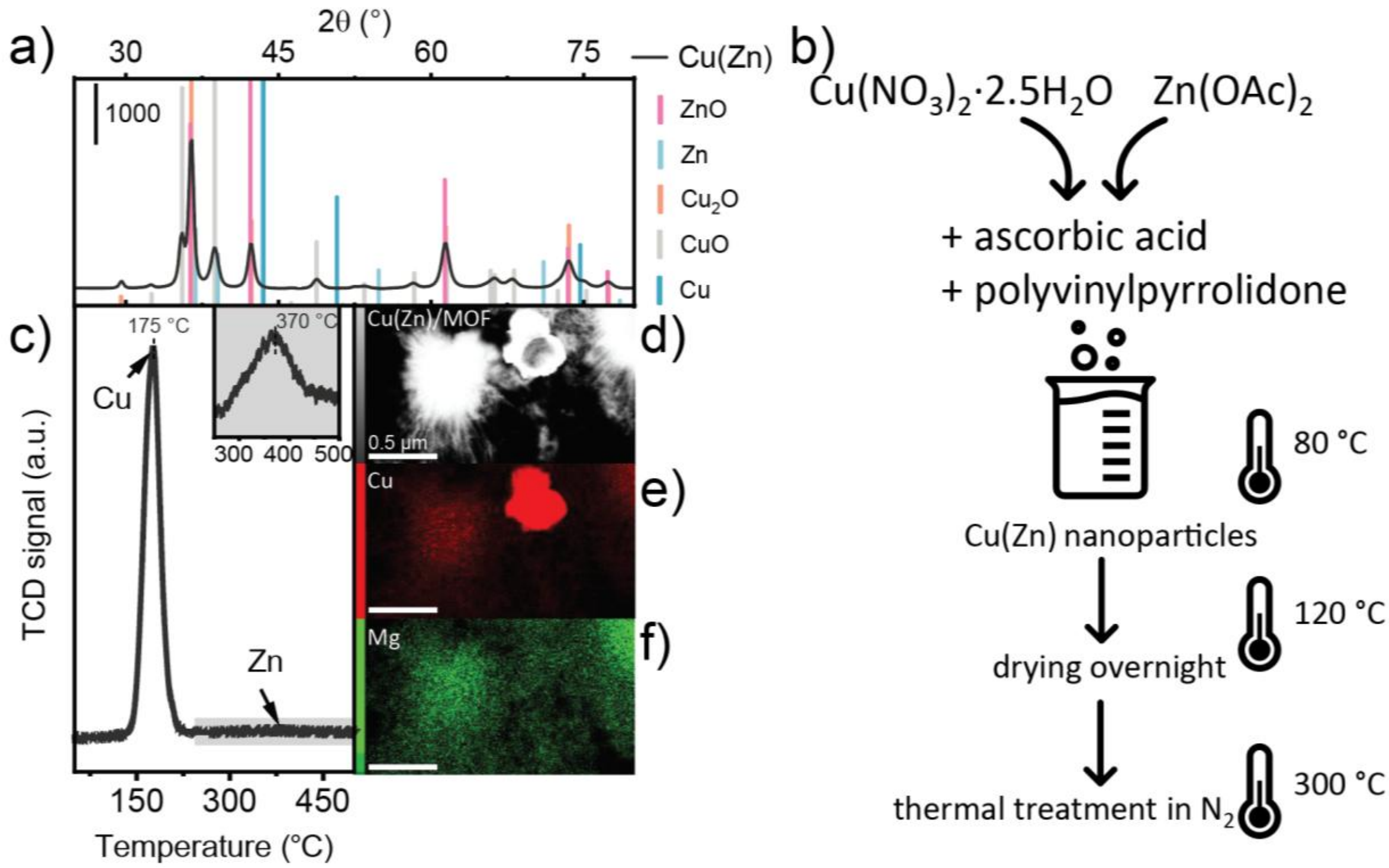


**Figure S4.** Cu(Zn) species and their properties. a) XRD of the Cu(Zn) with Zn, ZnO, Cu, $Cu_2O$, CuO as references; b) Scheme of the synthesis of the Cu(Zn) species. The XRD data were collected at 64 keV and transformed to Cu Kα wavelength; c) $H_2$-TPR of the Cu(Zn) species; (d-f) STEM-EDX of the Cu(Zn)/$Mg_2$(olz), Cu highlighted in red and Mg from the $Mg_2$(olz) is highlighted in green. Because Cu-based TEM grids were used for sample preparation, a minor contribution from the supporting grid may overlap with the Cu signal in the EDX maps. Scale bar corresponds to 500 nm.

**Procedure:** Cu(Zn) nanoparticles were synthesized by chemical reduction following a literature procedure [9], using $Cu(NO_3)_2·2.5H_2O$ (Sigma Aldrich, 98%) and $Zn(OAc)_2$ (Sigma Aldrich, 99.99%) as precursors, L-ascorbic acid (Sigma Aldrich, 99%) as a reducing agent, and

polyvinylpyrrolidone (PVP40, Sigma Aldrich) as a stabilizer in aqueous medium. The reaction was performed at 80 °C for 2 h, yielding a dark dispersion. The particles were recovered by centrifugation, dried at 120 °C, and thermally treated in air at 300 °C.

Cu(Zn)/$Mg_2$(olz) was prepared by wet impregnation of the MOF support with pre-synthesized Cu(Zn) species. The impregnation was performed in an inert-atmosphere glovebox using anhydrous acetonitrile (Sigma-Aldrich, 99.8%) to minimize adsorption of atmospheric moisture. The resulting suspension was stirred overnight at room temperature, followed by drying overnight at 60 °C under vacuum. The dried material was subsequently thermally treated at 200 °C using a Schlenk line to facilitate the removal of residual acetonitrile.

The same preparation procedure was subsequently applied to $Mg_2$(dobpdc) to obtain Cu(Zn)/$Mg_2$(dobpdc), which was used as a reference material for the in situ DRIFTS experiments.

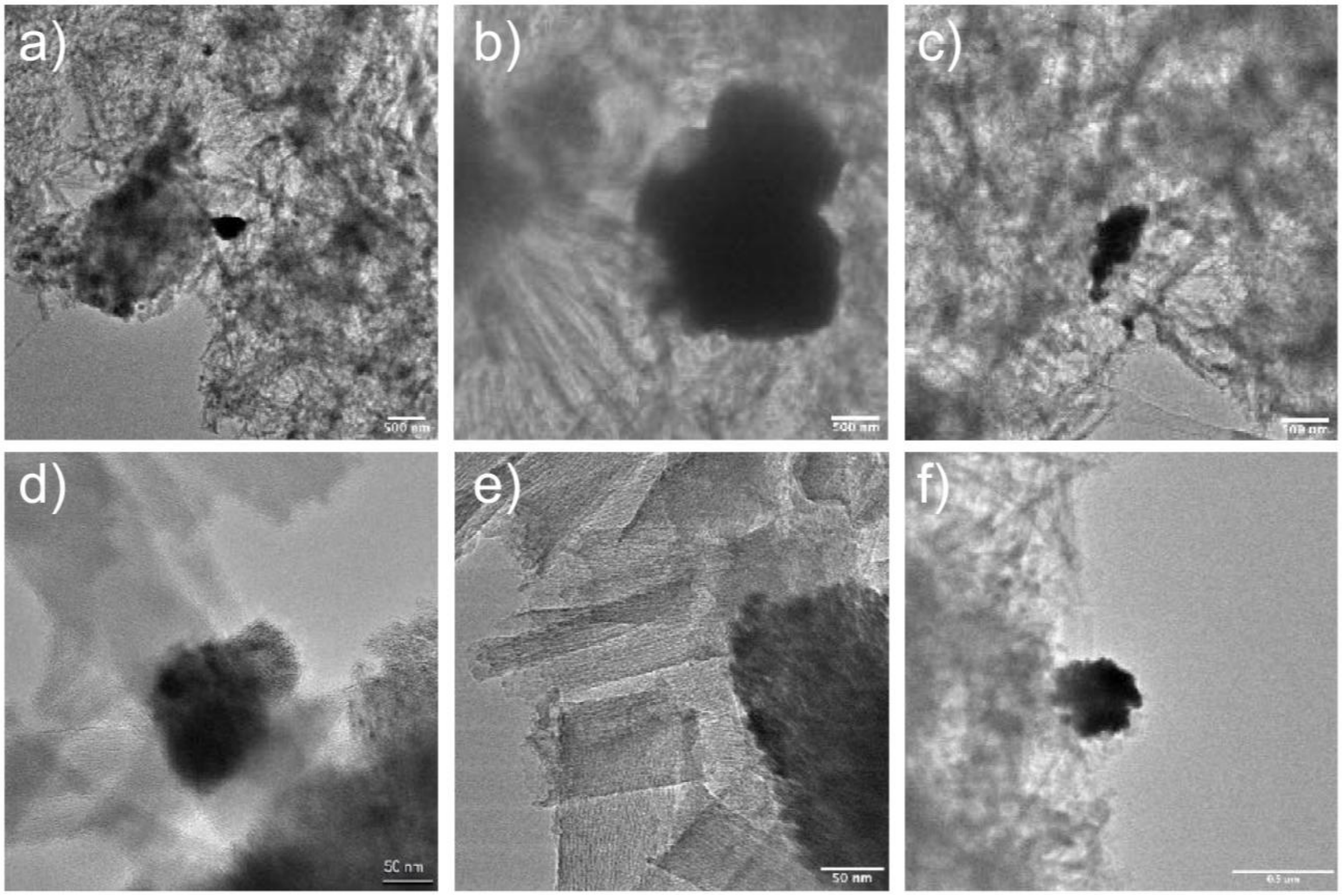


**Figure S5.** TEM images of the Cu(Zn)/$Mg_2$(olz) catalyst at various magnifications (a–f), showing additional examples of Cu(Zn) species associated with the $Mg_2$(olz) MOF matrix. The Cu(Zn) species appear as large, high-contrast features consistently co-localized with the sheet-like MOF structures, indicating their preferential association with the MOF sheets.

## 6. Catalytic testing in batch, liquid phase

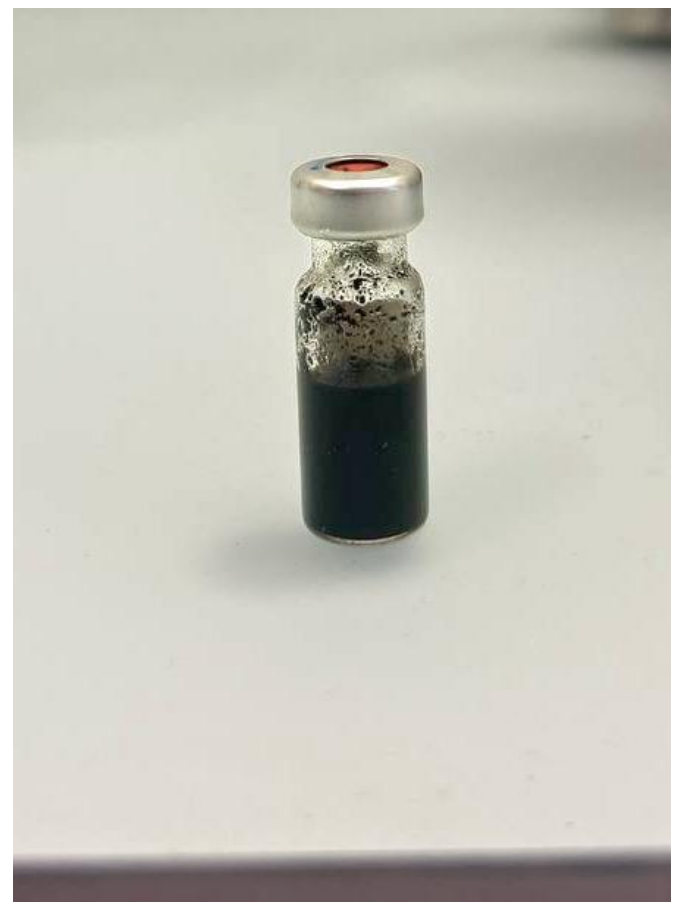

**Figure S6.** Cu(Zn)/$Mg_2$(olz) catalyst suspended in dioxane prior to the catalytic test in batch illustrating the suspension of the $Mg_2$(olz)-based catalyst.

**Table S2.** Catalyst performance metrics for low-temperature $CO_2$ hydrogenation to methanol compiled from literature and this work. Reported values include reaction temperature, pressure, and methanol space-time yield (STY). Data are reproduced from ref [10].

| **Catalyst** | Phase | T, °C | P, bar | STY, mmol/(g · h) | Reference |
|---|---|---|---|---|---|
| Cu(Zn)/$Mg_2$(olz) | g-l-s * | 150 | 52 | 2.8 (per g Cu+Zn) | This work |
| Cu(Zn)/$Mg_2$(olz) | g-l-s | 180 | 52 | 1.9 (per g Cu+Zn) | |
| CZA | g-l-s | 150 | 52 | 2.1 (per g Cu+Zn) | |
| CZA | g-l-s | 180 | 52 | 2.8 (per g Cu+Zn) | |
| Cu/ZnO | g-l-s | 180 | 60 | 0.3 | [11] |
| CuZnO-MOF-74-350 | g-s** | 190 | 40 | 2.5 | [12] |
| Cu/ZnO/$Al_2O_3$/ $ZrO_2$-RHT-9 | g-s | 190 | 50 | 2.7 | [13] |
| Cu/$ZrO_2$/CNFs/ZnO | g-l-s | 180 | 30 | 1.4 | [14] |
| $Er_{0.2}$CuZnO | g-s | 170 | 50 | 1.4 | [15] |
| $Pt_1$@MIL*** | g-l-s | 150 | 32 | 1.2 | [16] |
| Cu–$N_4$ | g-l-s | 150 | 32 | 4.2 | [17] |
| Cu/$ZrO_2$(20CZ-OG) | g-s | 200 | 40 | 5.6 | [18] |
| $Co_4N$ nanosheets | g-l-s | 150 | 32 | 70 | [19] |

* g-l-s – gas-liquid-solid; ** g-s – gas-solid; *** – MOF-based.

## 7. In situ DRIFTS under high-pressure $CO_2$ hydrogenation

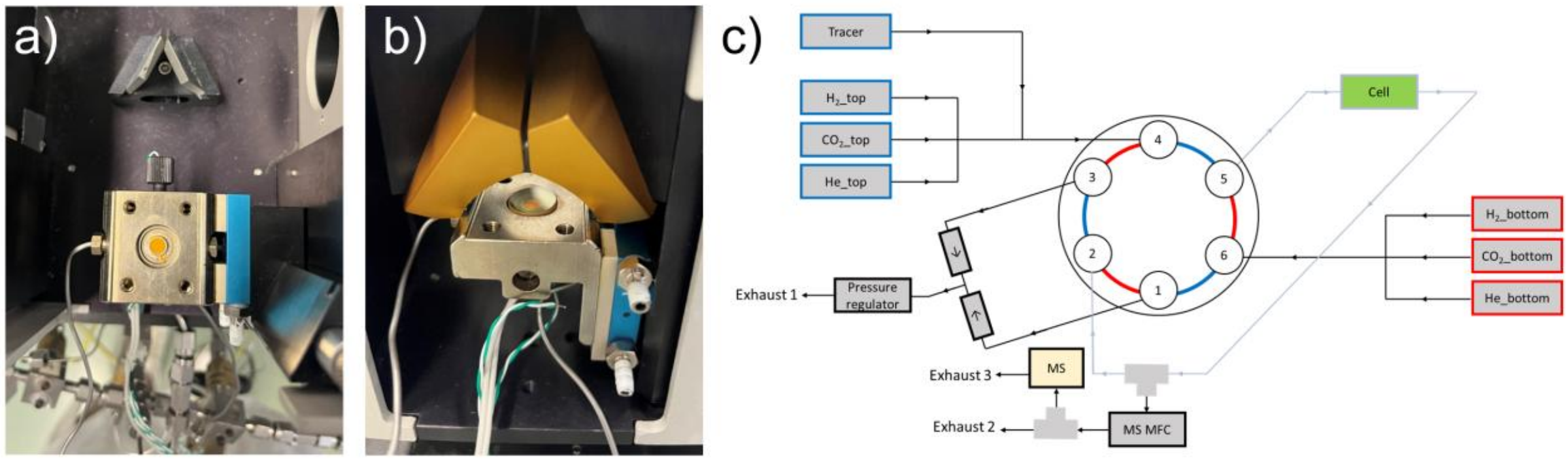


**Figure S7.** DRIFTS experimental setup for high-pressure measurements. (a) In situ DRIFTS cell with a catalyst loaded (top view); (b) sealed cell mounted in the spectrometer (side view); (c) schematic of the gas delivery and switching system used for high-pressure operation provided by Operando Group, Center for Energy and Environmental Sciences, Paul Scherrer Institute PSI.[20]

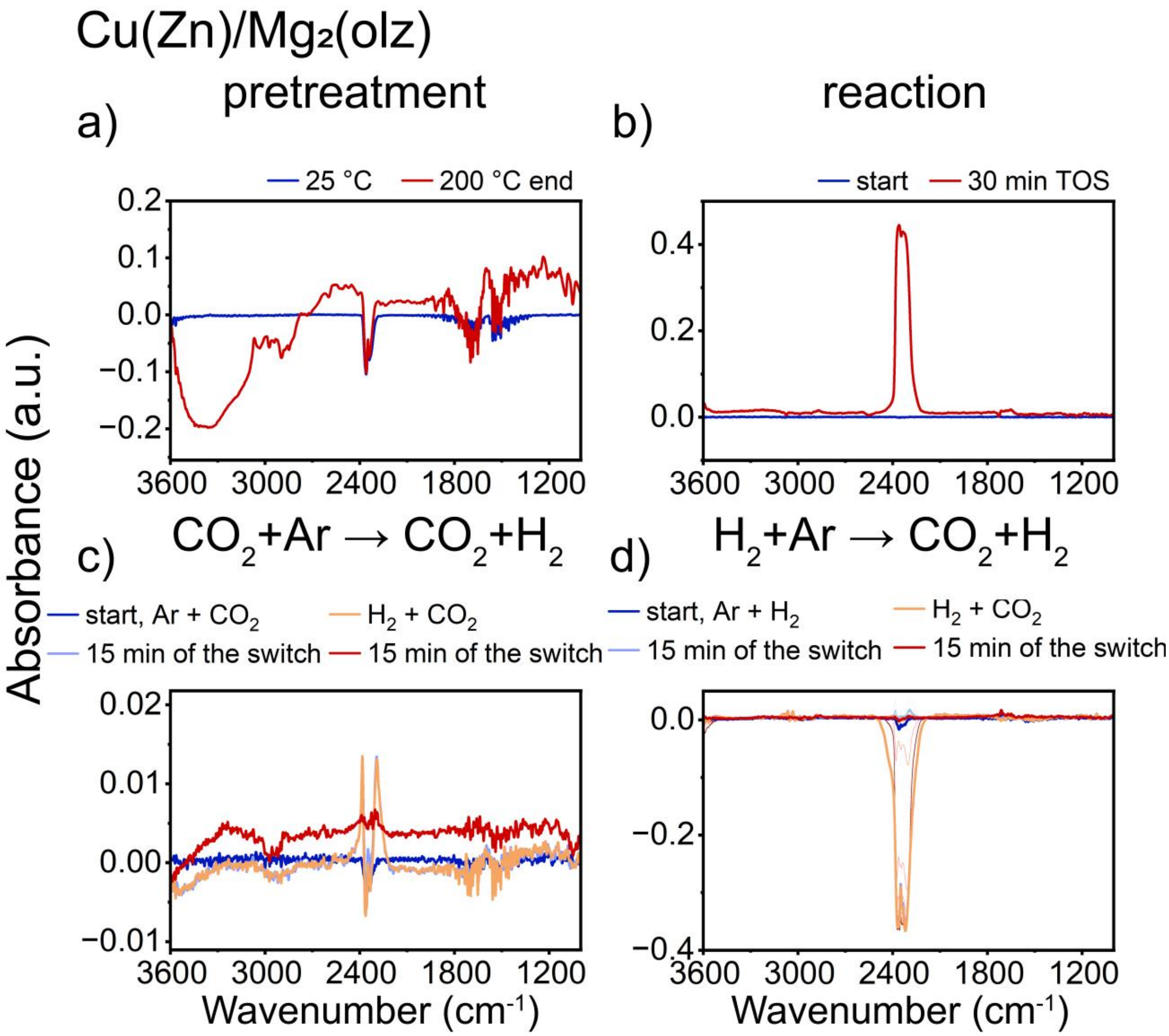


**Figure S8**. Overview of DRIFTS spectra recorded over Cu(Zn)/$Mg_2$(olz). (a) Pretreatment under $H_2$ + Ar during heating to 200 °C; (b) reaction conditions ($CO_2$ + $H_2$); (c) transient switching from $CO_2$ + Ar to $CO_2$ + $H_2$; (d) transient switching from $H_2$ + Ar to $CO_2$ + $H_2$. Conditions: 20 bar, $CO_2$:$H_2$ 1:3 (or Ar as a replacement gas), 200 °C pretreatment, 180 °C reaction, 15 mL/min flows.

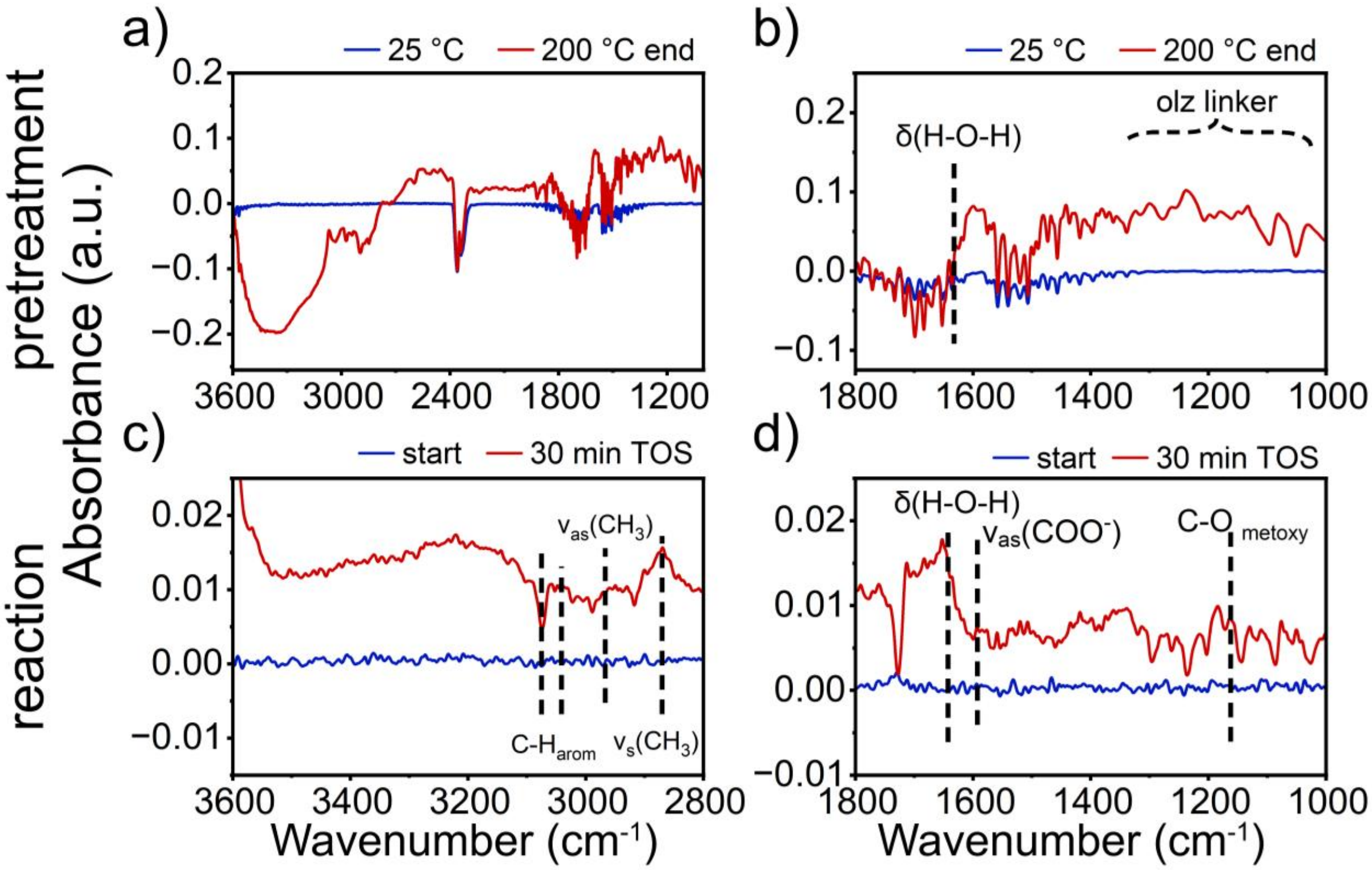


**Figure S9.** In situ DRIFTS spectra recorded over Cu(Zn)/$Mg_2$(olz) under $CO_2$ hydrogenation conditions. (a-b) Pretreatment (Ar + $H_2$) and (c-d) reaction. Spectra are shown in selected regions: O-H stretching + C-H stretching (a, c, 3600-2800 $cm^{-1}$) and the fingerprint region (b, d, 1800-1000 $cm^{-1}$). Conditions: 20 bar, $CO_2$:$H_2$ 1:3 (or Ar as a replacement gas), 200 °C pretreatment, 180 °C reaction, 15 mL/min flows.

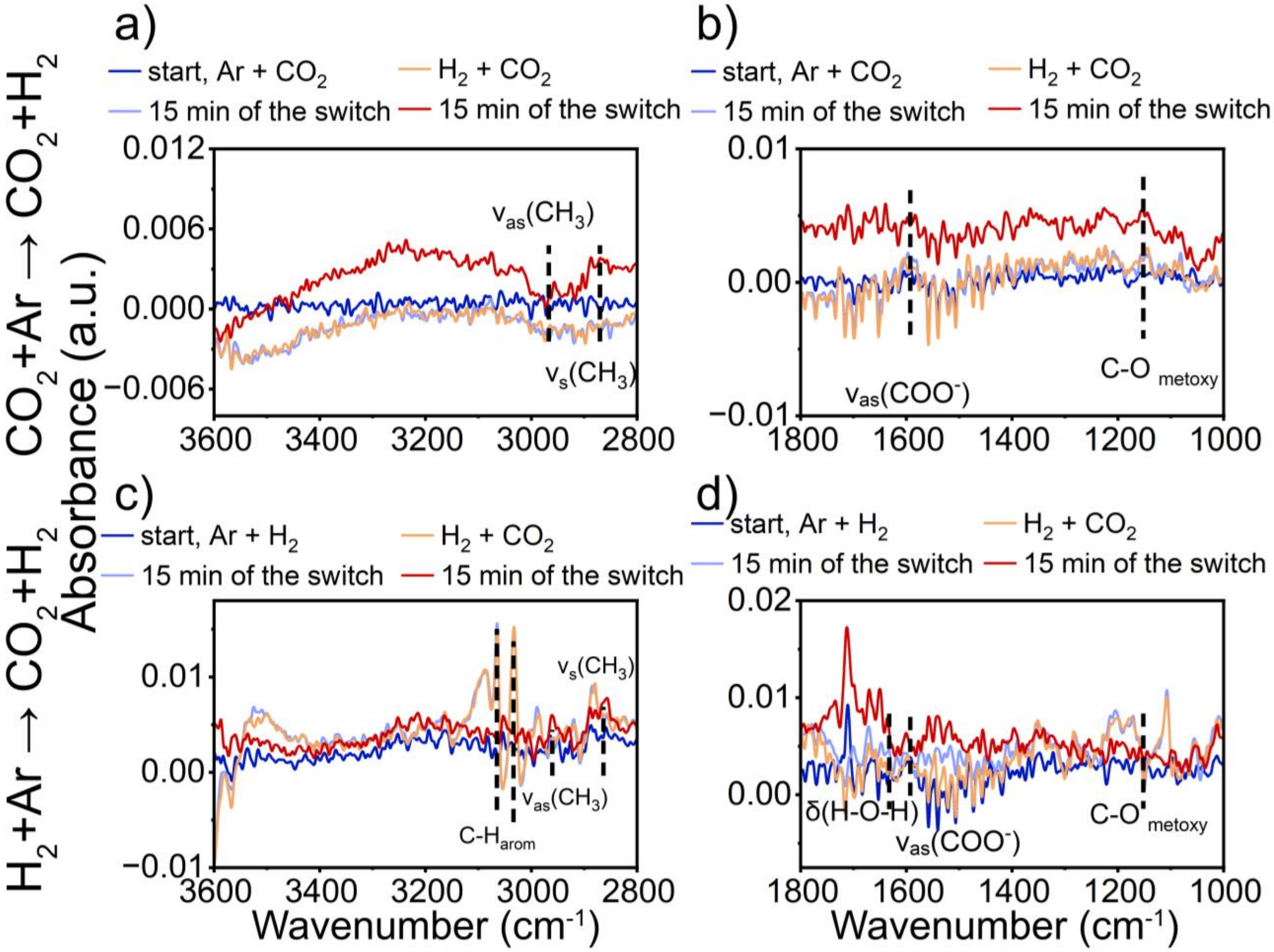


**Figure S10.** In situ DRIFTS spectra recorded over Cu(Zn)/$Mg_2$(olz) under $CO_2$ hydrogenation conditions. (a-b) Transient switching to $H_2$-free feed ($CO_2$ + Ar) and back to the reaction feed; (c-d) transient switching to $CO_2$-free feed ($H_2$ + Ar) and back. Spectra are shown in selected regions: O-H stretching + C-H stretching (a, c, 3600-2800 $cm^{-1}$) and the fingerprint region (b, d, 1800-1000 $cm^{-1}$). Conditions: 20 bar, $CO_2$:$H_2$ 1:3 (or Ar as a replacement gas), 200 °C pretreatment, 180 °C reaction, 15 mL/min flows.

### 7.1 Cu(Zn)/$Mg_2$(dobpdc) material for the DRIFTS comparison

**Synthesis of $Mg_2$(dobpdc)**: $Mg_2$(dobpdc)-based catalyst was synthesized as a reference material to assess whether the $Mg_2$(olz) contributes to the formation of reaction intermediates observed in

the DRIFTS measurements. Both $Mg_2$(olz) and $Mg_2$(dobpdc) belong to the MOF-74-II family, but differ in the organic linker, with dobpdc (4,4′-dioxidobiphenyl-3,3′-dicarboxylate) providing a shorter linker and slightly smaller pore size compared to the olz-based framework.

We followed a solvothermal procedure adapted from literature.[21],[22] $H_4$(dobpdc) (Chemie Brunschwig AG, 98%) and $Mg(NO_3)_2\cdot 6H_2O$ were dissolved in a 55:45 (v/v) methanol/DMF mixture, transferred to a flask, and heated at 120 °C for 20 h. The resulting white solid was isolated by filtration, washed with DMF and methanol, and solvent-exchanged in methanol. The material was activated several days under dynamic vacuum at 250 °C to yield $Mg_2$(dobpdc) as a white powder.

**Cu(Zn) incorporation**: The Cu(Zn)/$Mg_2$(dobpdc) catalyst was prepared analogously to the olz-based material by mixing pre-synthesized Cu(Zn) species with the activated MOF in dry acetonitrile in an air-free atmosphere, targeting comparable Cu(Zn) loadings. After that, the sample was re-activated under dynamic vacuum at 200 °C over the weekend.

Elemental composition (ICP). $Mg_2$(dobpdc): 26.1 wt% Mg; Cu(Zn)/$Mg_2$(dobpdc): 27.7 wt% Mg, 3.4 wt% Cu, 0.3 wt% Zn.

Textural properties. $N_2$ physisorption measurements show a decrease in surface area and pore volume upon Cu(Zn) incorporation; values for pristine and Cu(Zn)-loaded $Mg_2$(dobpdc): $S_{BET}$ 2530 $m^2/g$ and 1880 $m^2/g$; $V_{total}$ 1.3 $cm^3/g$ and 0.9 $cm^3/g$, respectively.

Powder XRD demonstrates preservation of the $Mg_2$(dobpdc) framework after Cu(Zn) incorporation (Fig. S11), with no major structural changes observed.

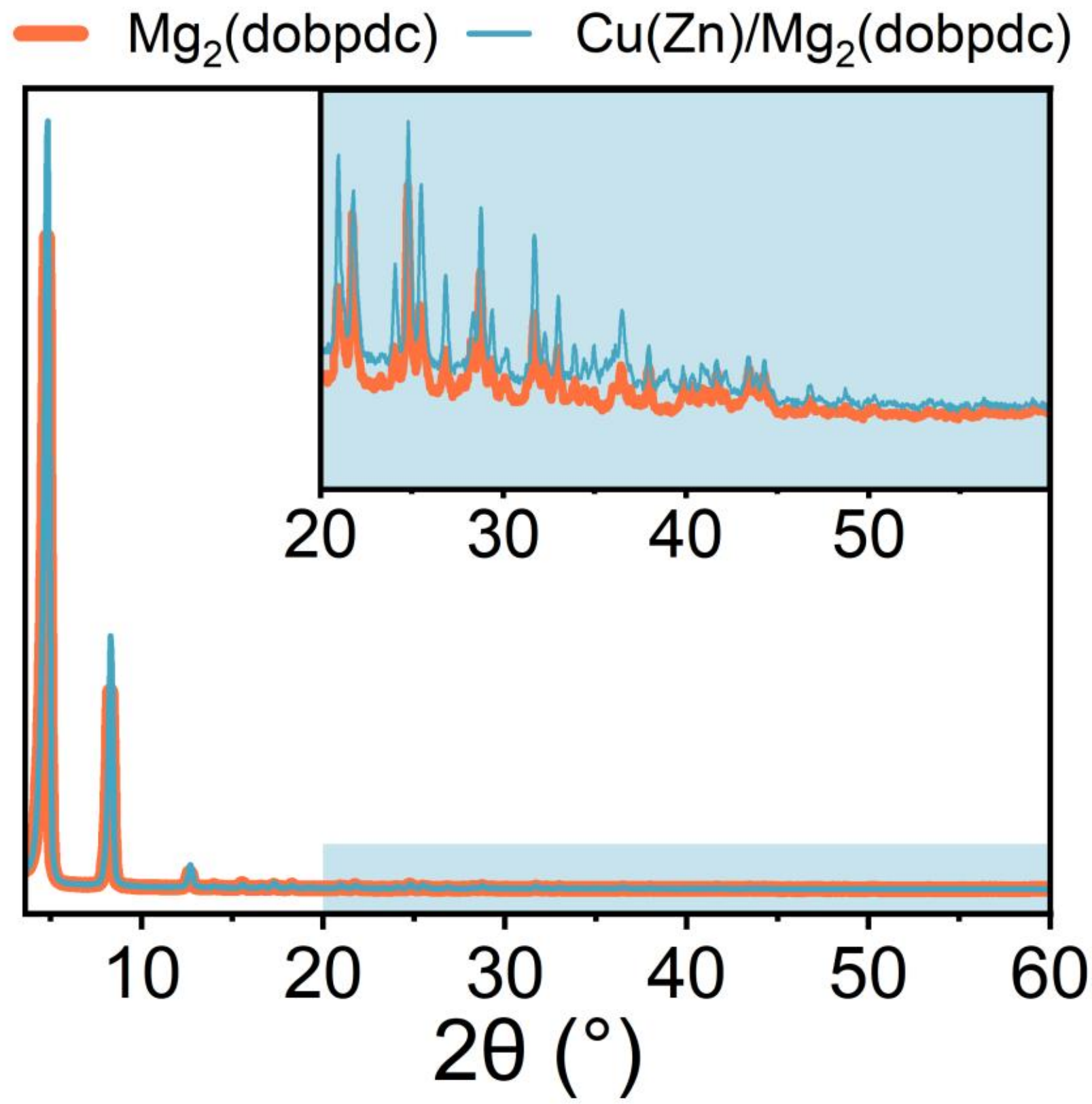


**Figure S11.** Powder XRD patterns of $Mg_2$(dobpdc) and Cu(Zn)/$Mg_2$(dobpdc) collected at a laboratory Cu K-alpha source, showing preservation of the MOF framework after Cu(Zn) incorporation. The inset shows an enlarged view of the XRD patterns at higher angles.

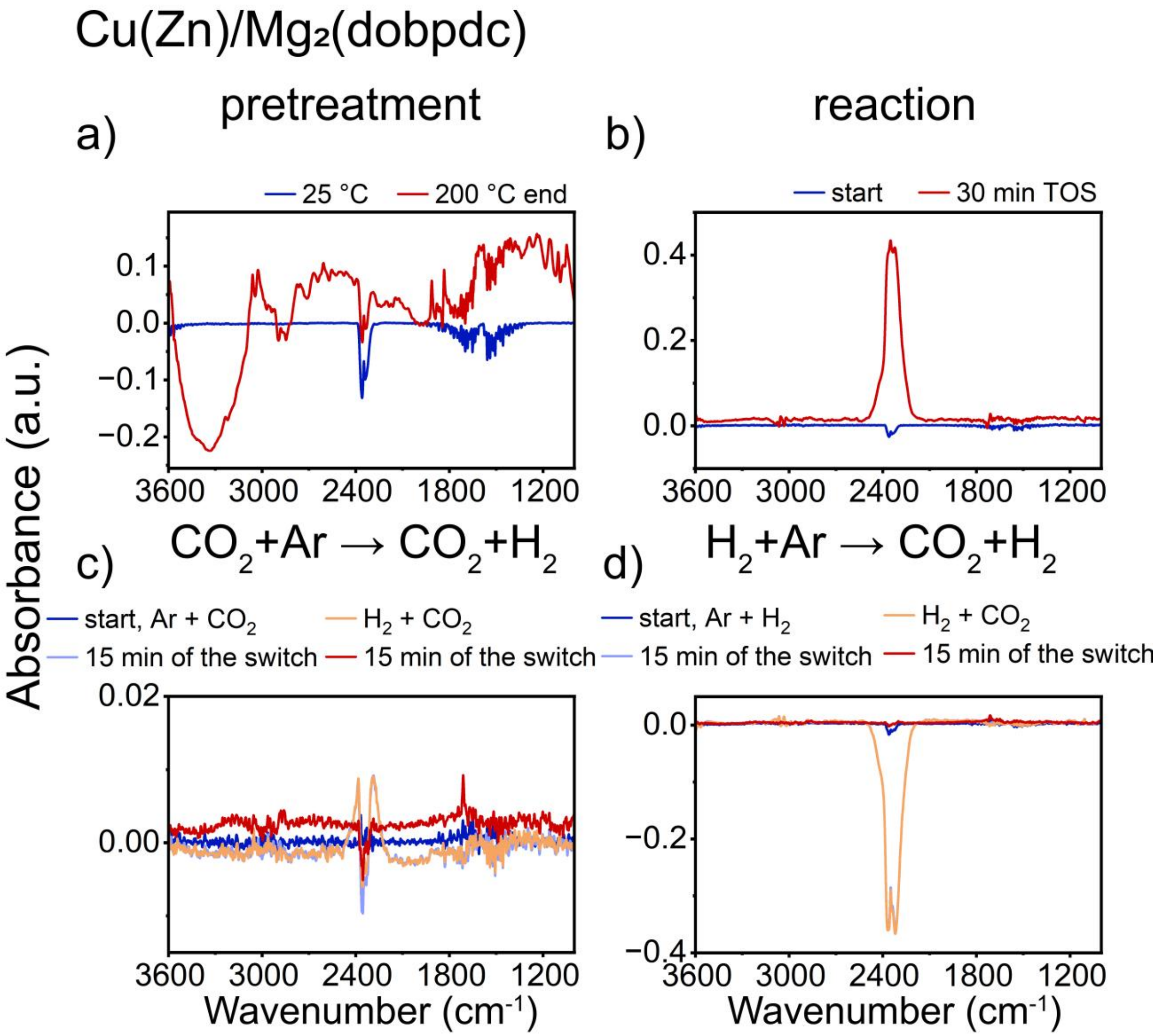


**Figure S12**. Overview of DRIFTS spectra recorded over Cu(Zn)/$Mg_2$(dobpdc). (a) Pretreatment under $H_2$ + Ar during heating to 200 °C; (b) reaction conditions ($CO_2$ + $H_2$); (c) transient switching from $CO_2$ + Ar to $CO_2$ + $H_2$; (d) transient switching from $H_2$ + Ar to $CO_2$ + $H_2$. Conditions: 20 bar, $CO_2$:$H_2$ 1:3 (or Ar as a replacement gas), 200 °C pretreatment, 180 °C reaction, 15 mL/min flows.

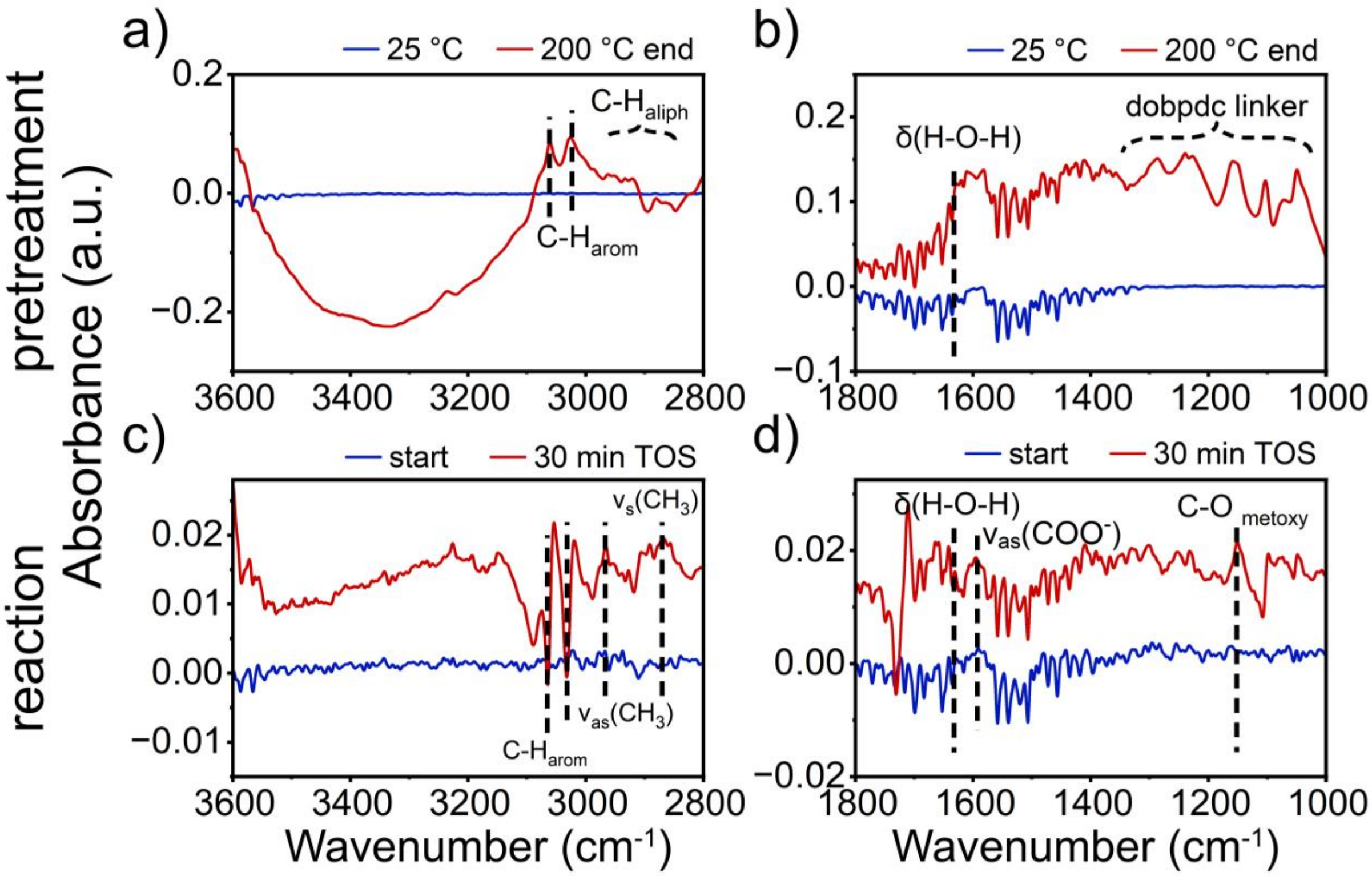


**Figure S13.** In situ DRIFTS spectra recorded over Cu(Zn)/$Mg_2$(dobpdc) under $CO_2$ hydrogenation conditions. (a-b) Pretreatment (Ar + $H_2$) and (c-d) reaction. Spectra are shown in selected regions: O-H stretching + C-H stretching (a, c, 3600-2800 $cm^{-1}$) and the fingerprint region (b, d, 1800-1000 $cm^{-1}$). Conditions: 20 bar, $CO_2$:$H_2$ 1:3 (or Ar as a replacement gas), 200 °C pretreatment, 180 °C reaction, 15 mL/min flows.

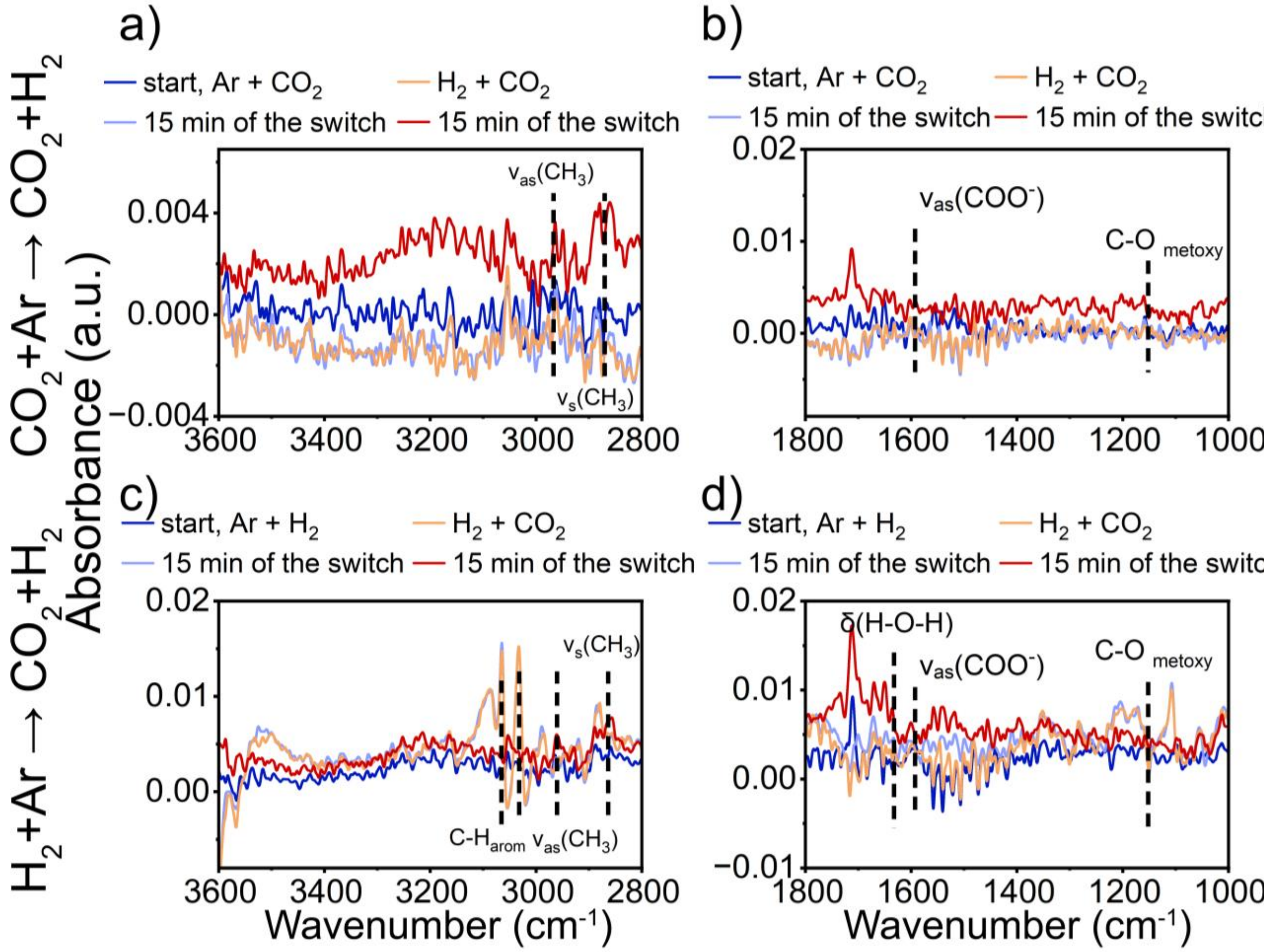


**Figure S14.** In situ DRIFTS spectra recorded over Cu(Zn)/$Mg_2$(dobpdc) under $CO_2$ hydrogenation conditions. (a-b) Transient switching to $H_2$-free feed ($CO_2$ + Ar) and back to the reaction feed; (c-d) transient switching to $CO_2$-free feed ($H_2$ + Ar) and back. Spectra are shown in selected regions: O-H stretching + C-H stretching (a, c, 3600-2800 $cm^{-1}$) and the fingerprint region (b, d, 1800-1000 $cm^{-1}$). Conditions: 20 bar, $CO_2$:$H_2$ 1:3 (or Ar as a replacement gas), 200 °C pretreatment, 180 °C reaction, 15 mL/min flows.

## 8. Quantification of $CO_2$ adsorption from the step-response experiments

Data collection: $CO_2$ concentration in the outlet gas was monitored as mol% versus time for both the $Mg_2$(olz) catalyst and an inert SiC reference. The experiment consisted of 30 min $CO_2$ + $H_2$ exposure followed by 30 min $N_2$ purge, at 40 bar total pressure and a $CO_2$:$H_2$ ratio of 1:30.

Blank subtraction: the SiC profile (blank) was subtracted from the MOF profile to remove contributions from system dead volume and non-specific adsorption.

Conversion to mol $CO_2$: each point in the concentration profile (mol%) was converted to mol $CO_2$ per minute using:

$$mol\ CO_2/\min = \frac{Q_{tot}\ (mL/min)}{22414\ mL/mol} \times \frac{mol\%\ CO_2}{100}$$

where $Q_{tot}$ is the total flow rate, 50 mL/min.

Integration over time: the difference between $Mg_2$(olz) and SiC mol $CO_2$/min profiles was integrated over the desorption period after the feed switch to $N_2$.

The integral was calculated as the sum of the average $CO_2$ molar flow between each time point multiplied by the time interval.

Normalization by catalyst mass: the total moles of $CO_2$ desorbed were divided by the catalyst mass (in g) to give mmol $CO_2$/g catalyst.

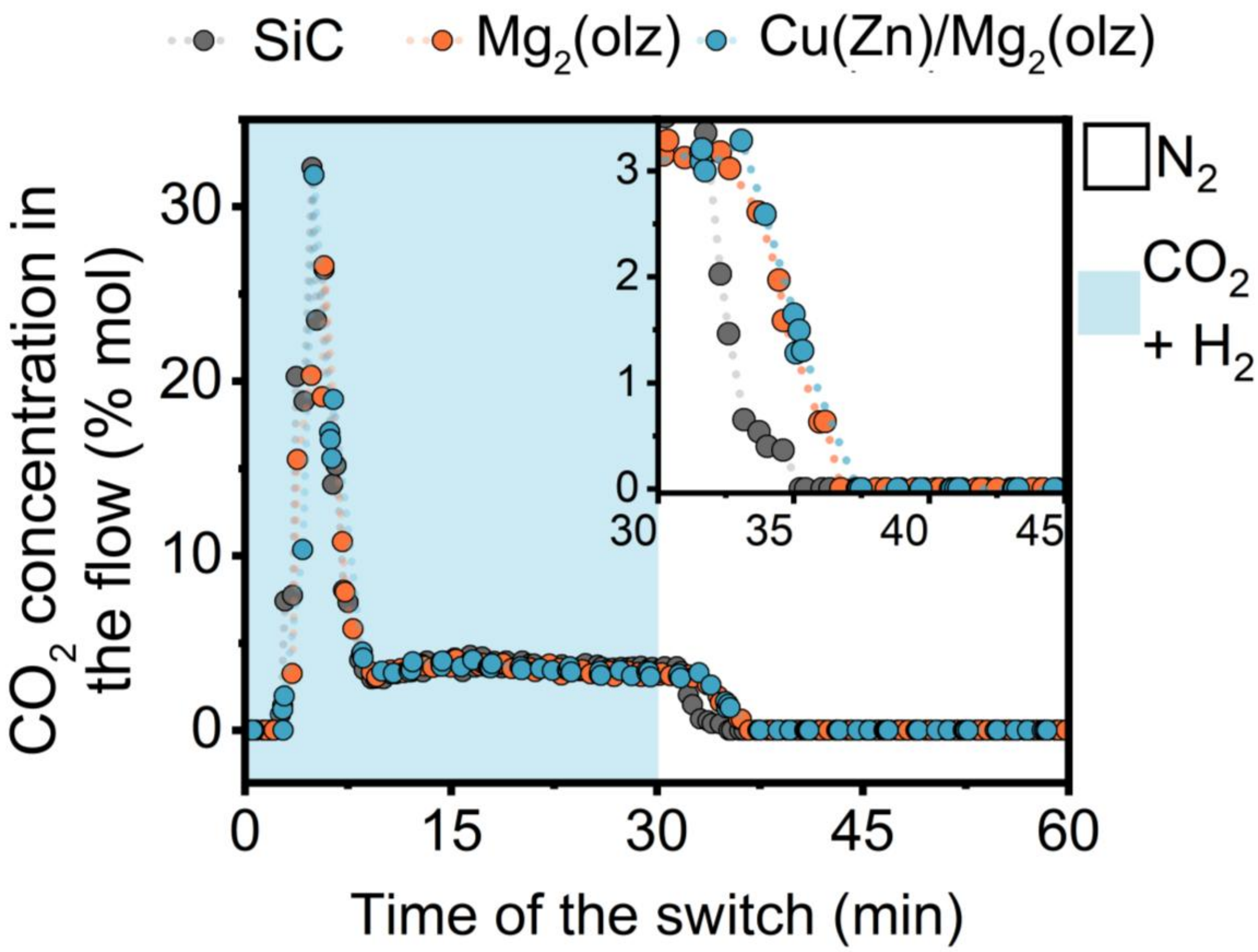


**Figure S15.** The $CO_2$ averaged response at 180 °C. Conditions: $CO_2$+$H_2$ / $N_2$ switching cycles, 30 min in $CO_2$+$H_2$ followed by 30 min in $N_2$, monitored via online gas chromatography in a plug-flow reactor at 40 bar. Feed composition $CO_2$:$H_2$ 1:30, total flow 50 mL/min, temperature range 30-180 °C, catalyst loading 30-40 mg for $Mg_2$(olz)-based samples and 100 mg for SiC.

## 9. $^1$H NMR spectra of the retained species after the step-response adsorption experiments

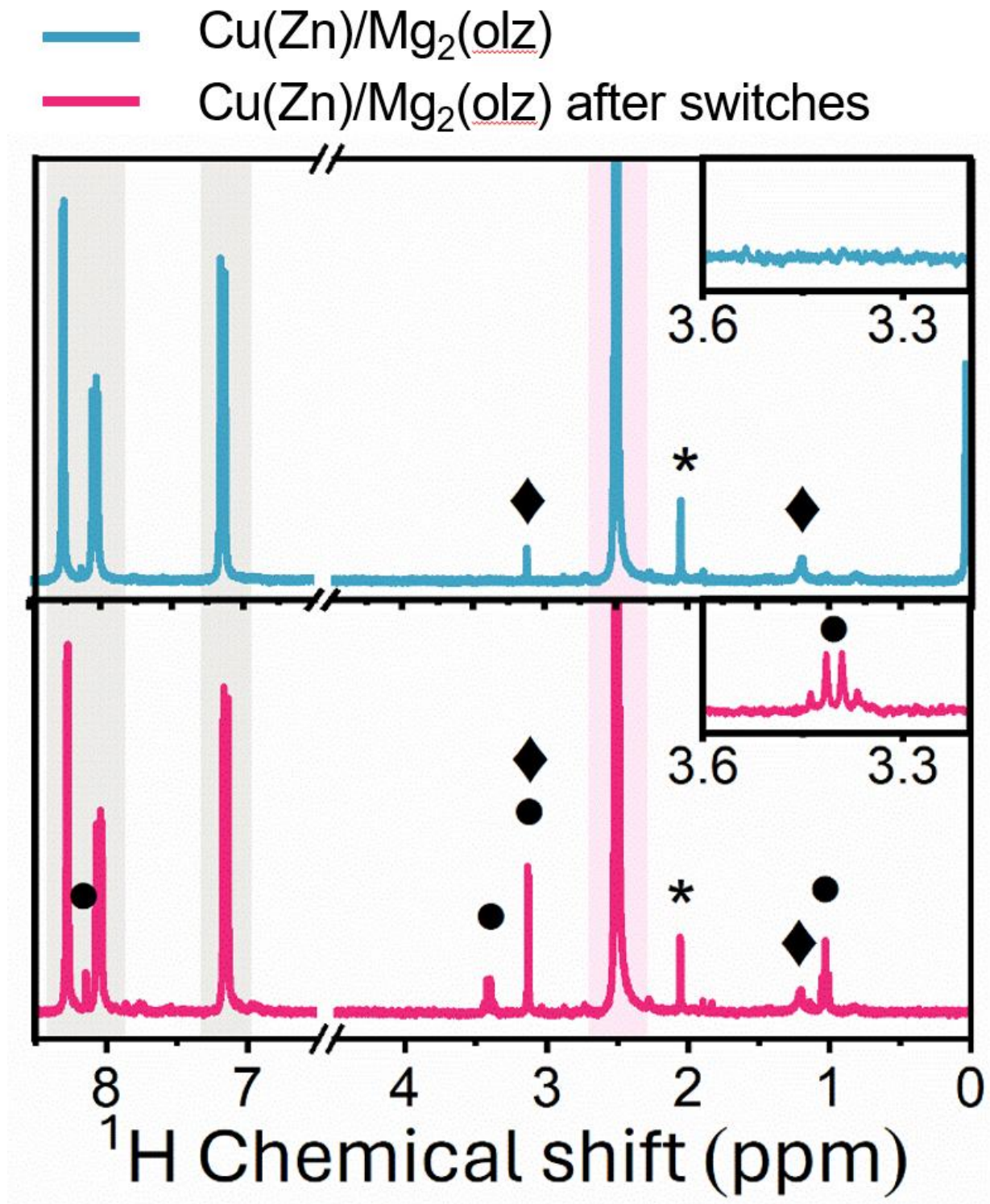


**Figure S16.** $^1$H NMR spectra of Cu(Zn)/$Mg_2$(olz) fresh and Cu(Zn)/$Mg_2$(olz) after the switches. The signals: $Mg_2$(olz) (grey highlight), DMSO (pink), possible methanol (insert), • oxygenates including formates and methoxy species, ♦ -$CH_3$ groups, * acetonitrile from WI.

## 10. State of Zinc in Cu(Zn)/$Mg_2$(olz) catalyst

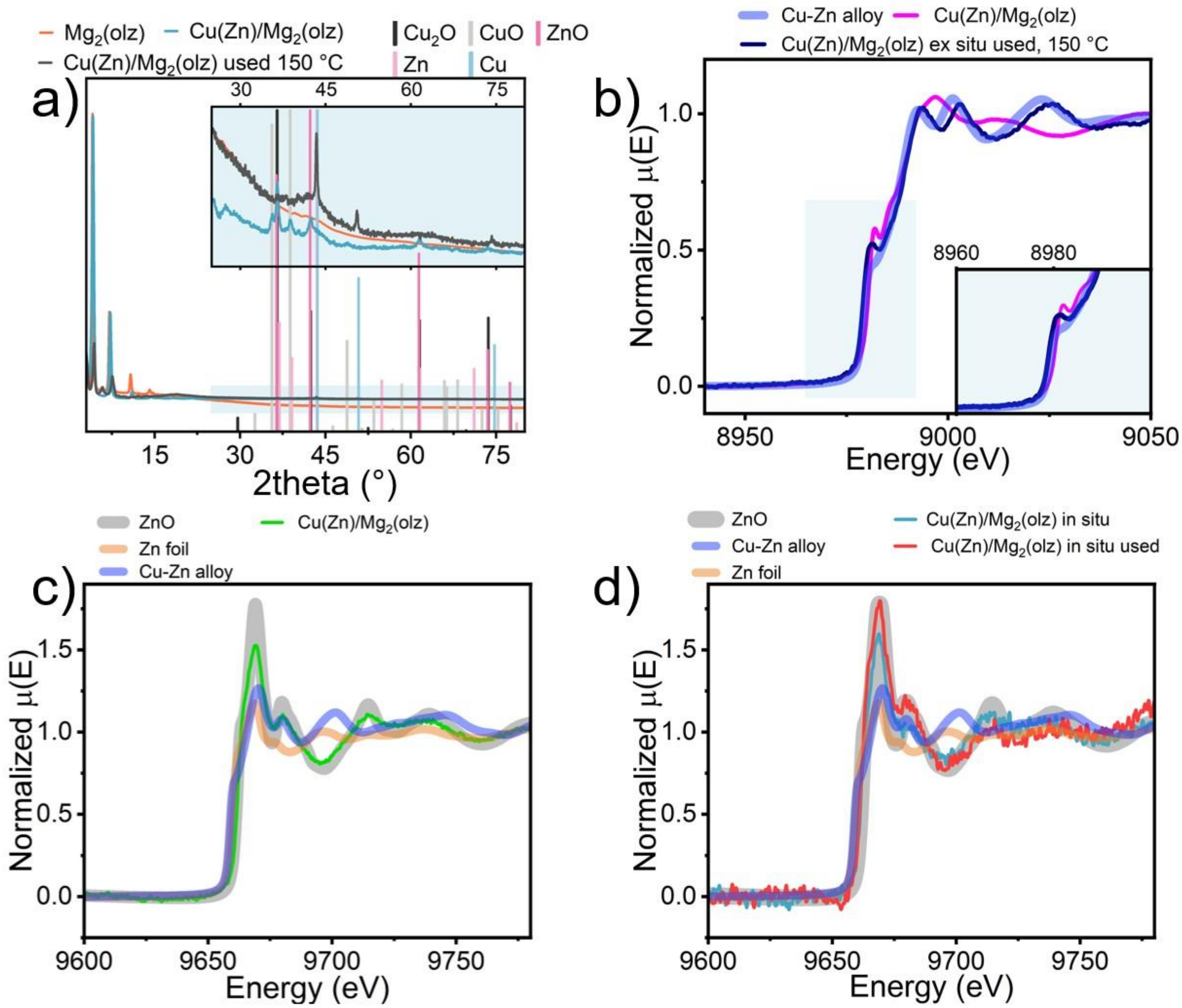


**Figure S17.** (a) Powder XRD patterns of pristine $Mg_2$(olz) - collected at ID15a ESRF; Cu(Zn)/$Mg_2$(olz) fresh, and Cu(Zn)/$Mg_2$(olz) after the batch reaction at 150 °C and 52 bar - collected at a laboratory Cu K-alpha source, with reference reflections of possible Cu- and Zn-containing phases. The inset highlights changes in the post-reaction diffraction pattern. (b) Cu K-edge XANES spectra of fresh and ex situ used Cu(Zn)/$Mg_2$(olz), compared with a Cu-Zn alloy reference. (c) Zn K-edge XANES spectrum of fresh Cu(Zn)/$Mg_2$(olz), compared with ZnO, metallic Zn, and Cu-Zn alloy references. (d) Zn K-edge XANES spectra of Cu(Zn)/$Mg_2$(olz)

measured before and after the in situ experiment, compared with the same references. The Cu-Zn alloy reference represents a metallic brass alloy (Cu-Zn solid solution). The Zn K-edge spectra predominantly resemble ZnO, with differences in spectral features potentially associated with particle size or local structural disorder rather than changes in Zn oxidation state.

### 11. XAS results - state of Copper in Cu(Zn)/$Mg_2$(olz) catalyst

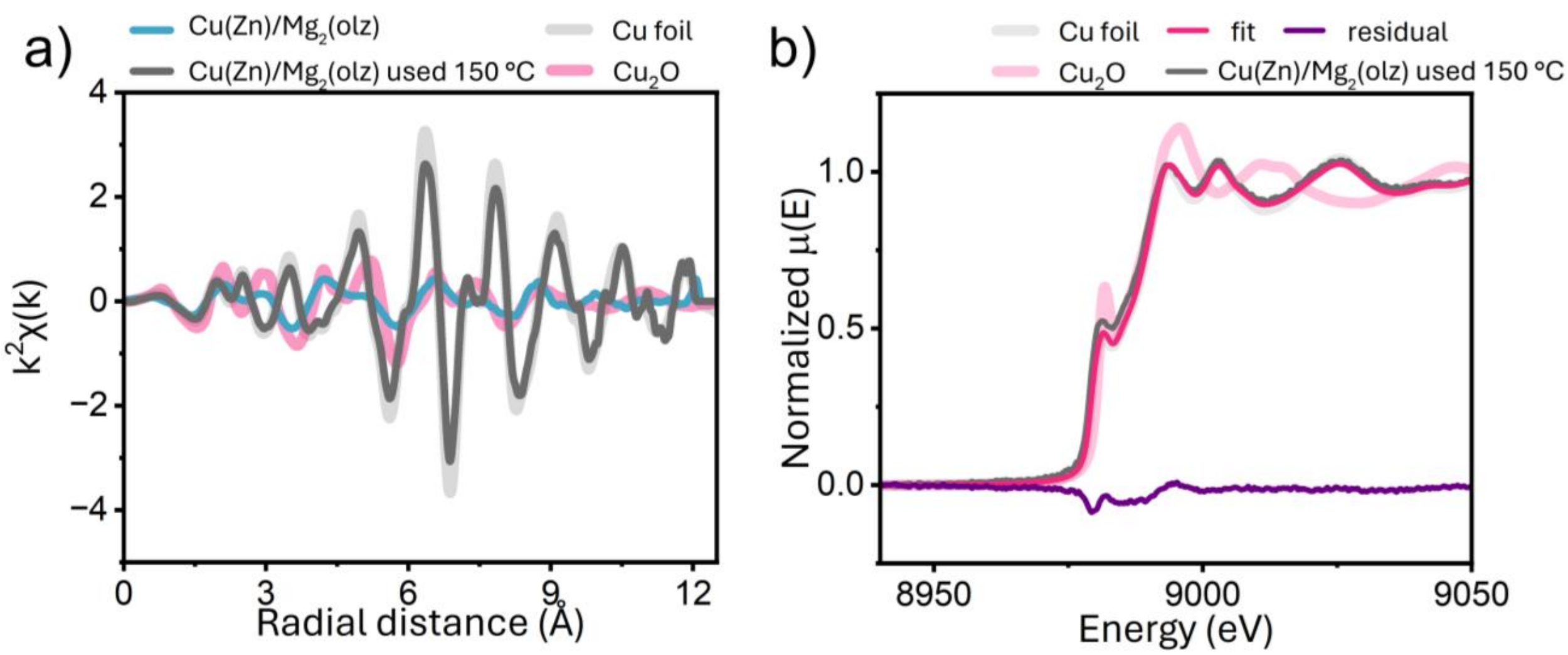


**Figure S18.** a) $k^2$-weighted EXAFS spectra of the Cu(Zn)/$Mg_2$(olz) catalyst compared with Cu foil and $Cu_2O$ references; b) XANES with linear combination fitting (LCF) and corresponding residuals for the used Cu(Zn)/$Mg_2$(olz) after testing at 150 °C. The fit indicates the coexistence of metallic Cu and minor $Cu_2O$ contributions. R-factor = 0.0043, reduced $\chi^2$ = 0.00073.

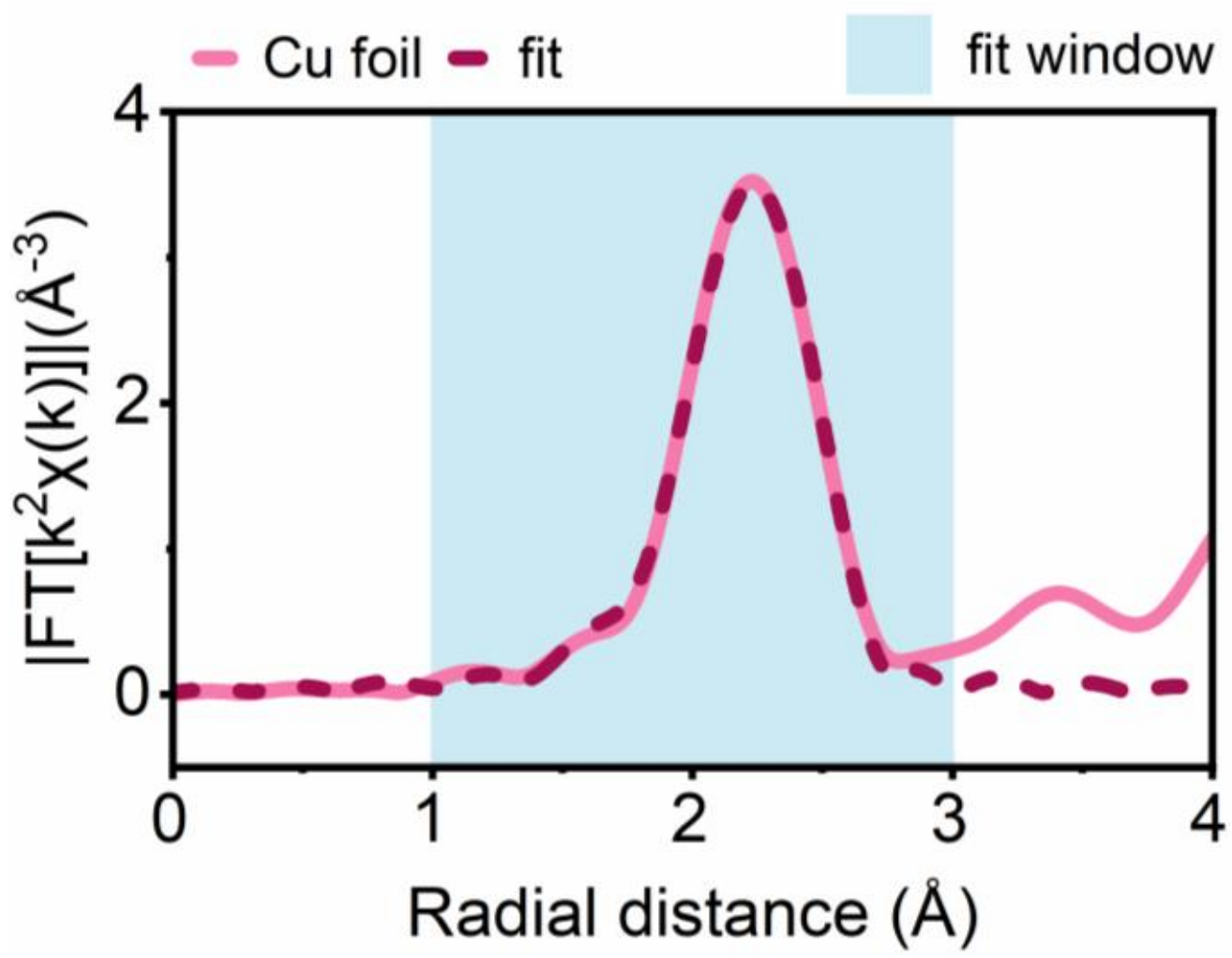


**Figure S19.** Averaged EXAFS spectra and their fit of the Cu foil.

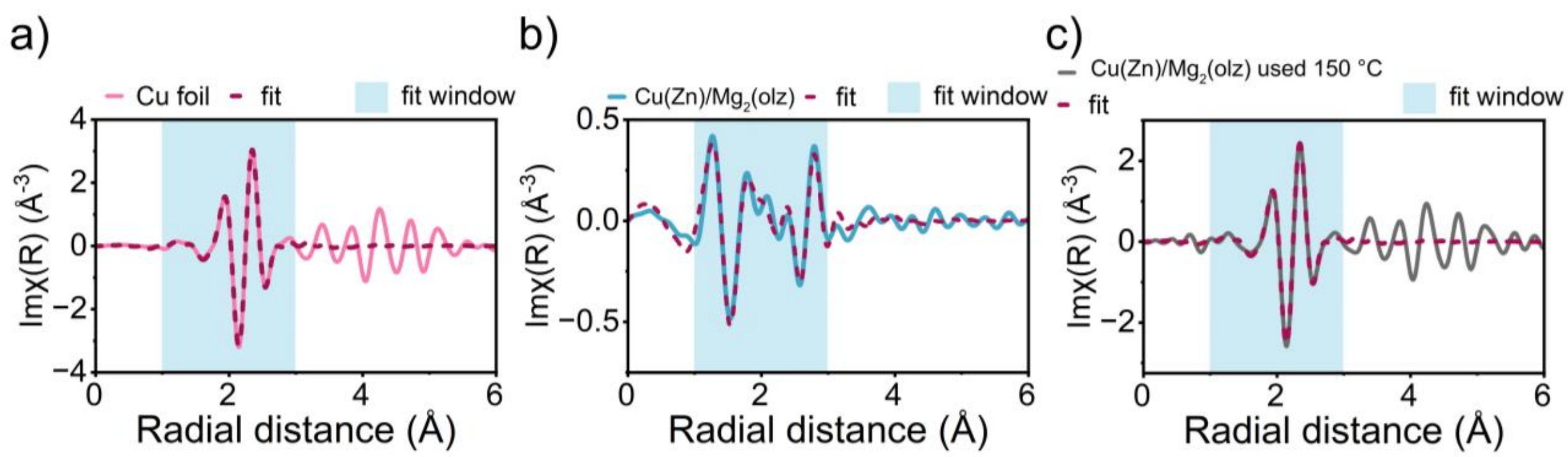


**Figure S20.** Experimental and best-fit curves of the imaginary part of the Cu K-edge EXAFS spectra in R-space for Cu foil, Cu(Zn)/$Mg_2$(olz), and Cu(Zn)/$Mg_2$(olz) used in batch at 150 °C. The shaded area indicates the fitting window.

The EXAFS analysis was performed at the Cu K-edge using $Cu_2O$ and metallic fcc Cu as structural models for Cu(Zn)/$Mg_2$(olz) and Cu(Zn)/$Mg_2$(olz) used in batch at 150 °C, respectively, based on the close similarity of their XANES features to Cu(I) and $Cu^0$ reference compounds.

The amplitude reduction factor $S_0^2$ was determined from a Cu metal foil by fitting the first Cu-Cu shell with a fixed coordination number of 12, yielding $S_0^2$=0.9. This value was subsequently

fixed for all sample fits. For the $Mg_2$(olz) MOF samples, the amplitude term was expressed as $S_0^2*N_i$, where $N_i$ is a scaling parameter refined during the fit. The effective coordination numbers ($N_{av}$) were calculated by multiplying $N_i$ by the coordination number of the corresponding bulk reference structure.

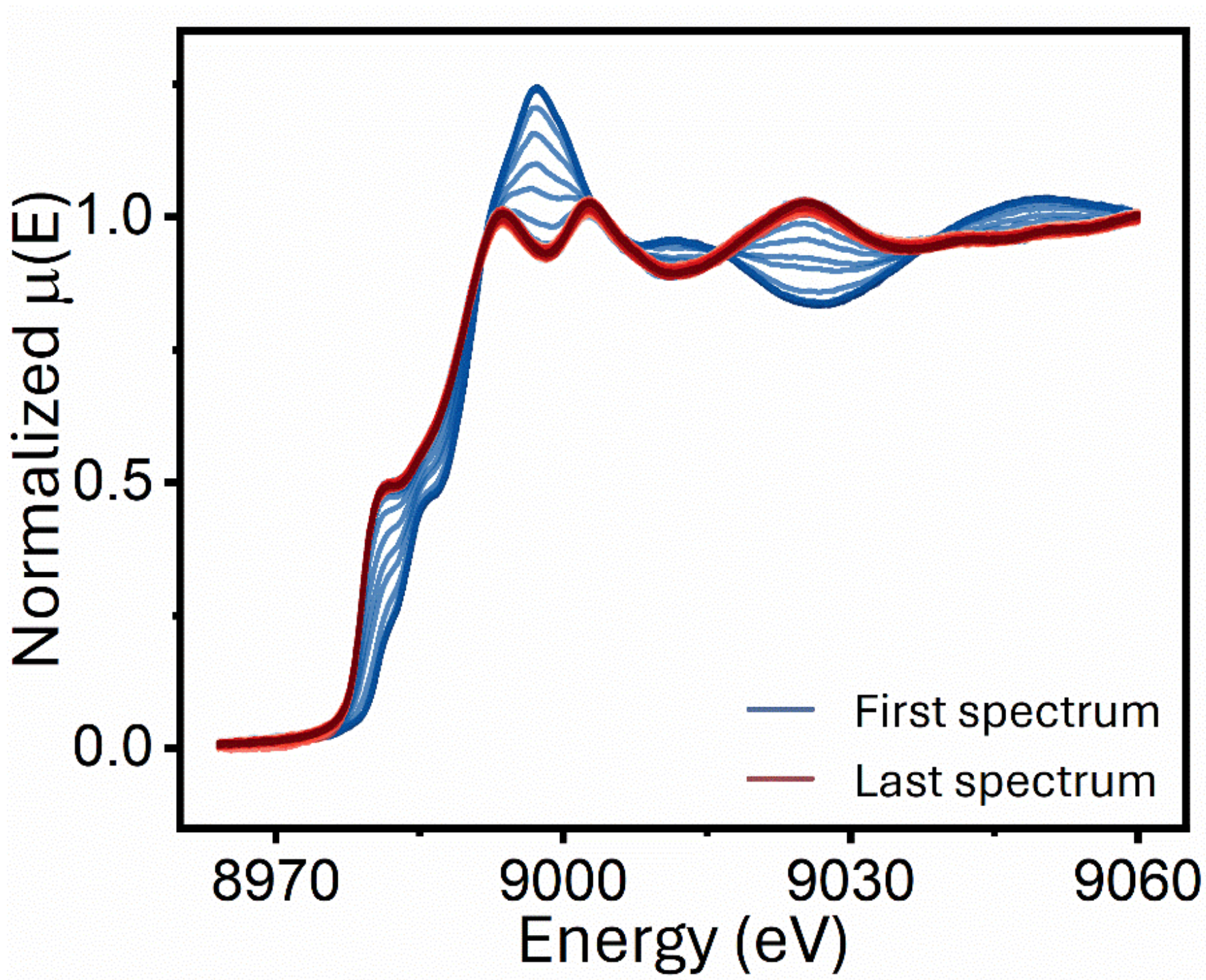


**Figure S21.** Normalized Cu K-edge XANES spectra collected during the in situ experiment over Cu(Zn)/$Mg_2$(olz). The first (blue) and last (red) spectra illustrate the evolution of copper species under reaction conditions. This dataset was used for the linear combination fitting (LCF) analysis.

## References


[1] G. B. M. Vaughan, R. Baker, R. Barret, J. Bonnefoy, T. Buslaps, S. Checchia, D. Duran, F. Fihman, P. Got, J. Kieffer, "ID15A at the ESRF–a beamline for high speed operando X-ray diffraction, diffraction tomography and total scattering" *J. Synchrotron Radiat.*

**2020**, *27*, 515–528.

[2] F. Cambiè, D. C. Cano Blanco, I. Kochetygov, L. Maggiulli, “Shedding synchrotron light on MOF-74 formation in aqueous phase,” European Synchrotron Radiation Facility, 2027, DOI: 10.15151/ESRF-ES-1566395715.

[3] A. Liutkova, H. Zhang, J. F. M. Simons, B. Mezari, M. Mirolo, G. A. Garcia, E. J. M. Hensen, N. Kosinov, “Ca Cations Impact the Local Environment inside HZSM-5 Pores during the Methanol-to-Hydrocarbons Reaction” *ACS Catal.* **2023**, *13*, 3471–3484.

[4] J. L. Alfke, M. Tejeda-Serrano, S. Phadke, A. Tereshchenko, T. Z. H. Gani, L. Rochlitz, S. B. X. Y. Zhang, L. Lin, C. Copéret, O. V Safonova, “Boundary Conditions for Promotion versus Poisoning in Copper–Gallium-Based CO2-to-Methanol Hydrogenation Catalysts” *ACS Catal.* **2024**, *14*, 9166–9175.

[5] O. Mathon, A. Beteva, J. Borrel, D. Bugnazet, S. Gatla, R. Hino, I. Kantor, T. Mairs, M. Muñoz, S. Pasternak, “The time-resolved and extreme conditions XAS (TEXAS) facility at the European Synchrotron Radiation Facility: the general-purpose EXAFS bending-magnet beamline BM23” *J. Synchrotron Radiat.* **2015**, *22*, 1548–1554.

[6] M. Newville, “Larch: An Analysis Package for XAFS and Related Spectroscopies,” *J. Phys.: Conf. Ser.* 2013, 430, 012007.

[7] Z. Zhu, S. T. Parker, A. C. Forse, J.-H. Lee, R. L. Siegelman, P. J. Milner, H. Tsai, M. Ye, S. Xiong, M. V Paley, A. A. Uliana, J. Oktawiec, B. Dinakar, S. A. Didas, K. R. Meihaus, J. A. Reimer, J. B. Neaton, J. R. Long, “Cooperative Carbon Dioxide Capture in Diamine-Appended Magnesium–Olsalazine Frameworks” *J. Am. Chem. Soc.* **2023**, *145*, 17151–17163.

[8] D. J. Levine, T. Runčevski, M. T. Kapelewski, B. K. Keitz, J. Oktawiec, D. A. Reed, J. A.

Mason, H. Z. H. Jiang, K. A. Colwell, C. M. Legendre, S. A. FitzGerald, J. R. Long, "Olsalazine-Based Metal–Organic Frameworks as Biocompatible Platforms for H2 Adsorption and Drug Delivery" *J. Am. Chem. Soc.* **2016**, *138*, 10143–10150.

[9] J. Xiong, Y. Wang, Q. Xue, X. Wu, "Synthesis of highly stable dispersions of nanosized copper particles using L-ascorbic acid" *Green Chem.* **2011**, *13*, 900–904.

[10] J. Wang, M. Ma, Q. Ma, T.-S. Zhao, "Low-temperature hydrogenation of CO2 to methanol: progress in constructing catalytic active sites" *Rev. Chem. Eng.* **2025**, *41*, 575–599.

[11] H. Nieminen, G. Givirovskiy, A. Laari, T. Koiranen, "Alcohol promoted methanol synthesis enhanced by adsorption of water and dual catalysts" *J. CO2 Util.* **2018**, *24*, 180–189.

[12] C. Han, H. Zhang, C. Li, H. Huang, S. Wang, P. Wang, J. Li, "The regulation of Cu-ZnO interface by Cu-Zn bimetallic metal organic framework-templated strategy for enhanced CO2 hydrogenation to methanol" *Appl. Catal. A Gen.* **2022**, *643*, 118805.

[13] S. Xiao, Y. Zhang, P. Gao, L. Zhong, X. Li, Z. Zhang, H. Wang, W. Wei, Y. Sun, "Highly efficient Cu-based catalysts via hydrotalcite-like precursors for CO2 hydrogenation to methanol" *Catal. Today* **2017**, *281*, 327–336.

[14] I. U. Din, M. S. Shaharun, A. Naeem, S. Tasleem, P. Ahmad, "Revalorization of CO2 for methanol production via ZnO promoted carbon nanofibers based Cu-ZrO2 catalytic hydrogenation" *J. Energy Chem.* **2019**, *39*, 68–76.

[15] C. Huang, S. Zhang, W. Wang, H. Zhou, Z. Shao, L. Xia, H. Wang, Y. Sun, "Modulation of Electronic Metal-Support Interaction between Cu and ZnO by Er for Effective Low-Temperature CO2 Hydrogenation to Methanol" *ACS Catal.* **2024**, *14*, 1324–1335.

[16] Y. Chen, H. Li, W. Zhao, W. Zhang, J. Li, W. Li, X. Zheng, W. Yan, W. Zhang, J. Zhu, "Optimizing reaction paths for methanol synthesis from CO2 hydrogenation via metal-ligand cooperativity" *Nat. Commun.* **2019**, *10*, 1885.

[17] T. Yang, X. Mao, Y. Zhang, X. Wu, L. Wang, M. Chu, C.-W. Pao, S. Yang, Y. Xu, X. Huang, "Coordination tailoring of Cu single sites on C3N4 realizes selective CO2 hydrogenation at low temperature" *Nat. Commun.* **2021**, *12*, 6022.

[18] H. Chang, F. Gao, A. Luo, Y. Liu, Y. Zhu, H. He, Y. Cao, "Oxygen vacancy promoted carbon dioxide activation over Cu/ZrO2 for CO2-to-methanol conversion" *Chem. Commun.* **2023**, *59*, 7647–7650.

[19] L. Wang, W. Zhang, X. Zheng, Y. Chen, W. Wu, J. Qiu, X. Zhao, X. Zhao, Y. Dai, J. Zeng, "Incorporating nitrogen atoms into cobalt nanosheets as a strategy to boost catalytic activity toward CO2 hydrogenation" *Nat. Energy* **2017**, *2*, 869–876.

[20] S. Phadke, "Deciphering Structure–Activity Relationships in Pd-Zn Catalysts for $CO_2$ Hydrogenation to Methanol through Advanced Synthesis and XAS Characterization," ETH Zurich, **2025**.

[21] T. M. McDonald, J. A. Mason, X. Kong, E. D. Bloch, D. Gygi, A. Dani, V. Crocella, F. Giordanino, S. O. Odoh, W. S. Drisdell, "Cooperative insertion of CO2 in diamine-appended metal-organic frameworks" *Nature* **2015**, *519*, 303–308.

[22] R. L. Siegelman, T. M. McDonald, M. I. Gonzalez, J. D. Martell, P. J. Milner, J. A. Mason, A. H. Berger, A. S. Bhown, J. R. Long, "Controlling cooperative CO2 adsorption in diamine-appended Mg2 (dobpdc) metal–organic frameworks" *J. Am. Chem. Soc.* **2017**, *139*, 10526–10538.
